# SENP3-FIS1 axis promotes mitophagy and cell survival under hypoxia

**DOI:** 10.1038/s41419-024-07271-8

**Published:** 2024-12-05

**Authors:** Alice Zhao, Laura Maple, Juwei Jiang, Katie N. Myers, Callum G. Jones, Hannah Gagg, Connor McGarrity-Cottrell, Ola Rominiyi, Spencer J. Collis, Greg Wells, Marufur Rahman, Sarah J. Danson, Darren Robinson, Carl Smythe, Chun Guo

**Affiliations:** 1 School of Biosciences, University of Sheffield, Firth Court, Western Bank, Sheffield, S10 2TN UK; 2 Division of Clinical Medicine, University of Sheffield Medical School, Sheffield, S10 2RX UK; 3 Division of Neuroscience, University of Sheffield Medical School, Sheffield, S10 2HQ UK; 4 Department of Neurosurgery, Sheffield Teaching Hospitals NHS Foundation Trust, Sheffield, S10 2JF UK; 5 Ex vivo Project Team, Division of Clinical Medicine, University of Sheffield Medical School, Sheffield, S10 2RX UK

**Keywords:** Mitophagy, Proteases

## Abstract

SUMOylation, the covalent attachment of the small ubiquitin-like modifier (SUMO) to target proteins, and its reversal, deSUMOylation by SUMO proteases like Sentrin-specific proteases (SENPs), are crucial for initiating cellular responses to hypoxia. However, their roles in subsequent adaptation processes to hypoxia such as mitochondrial autophagy (mitophagy) remain unexplored. Here, we show that general SUMOylation, particularly SUMO2/3 modification, suppresses mitophagy under both normoxia and hypoxia. Furthermore, we identify deSUMO2/3-ylation enzyme SENP3 and mitochondrial Fission protein 1 (FIS1) as key players in hypoxia-induced mitophagy (HIM), with SUMOylatable FIS1 acting as a crucial regulator for SENP3-mediated HIM regulation. Interestingly, we find that hypoxia promotes FIS1 SUMO2/3-ylation and triggers an interaction between SUMOylatable FIS1 and Rab GTPase-activating protein Tre-2/Bub2/Cdc16 domain 1 family member 17 (TBC1D17), which in turn suppresses HIM. Therefore, we propose a novel SUMOylation-dependent pathway where the SENP3-FIS1 axis promotes HIM, with TBC1D17 acting as a fine-tuning regulator. Importantly, the SENP3-FIS1 axis plays a protective role against hypoxia-induced cell death, highlighting its physiological significance, and hypoxia-inducible FIS1-TBC1D17 interaction is detectable in primary glioma stem cell-like (GSC) cultures derived from glioblastoma patients, suggesting its disease relevance. Our findings not only provide new insights into SUMOylation/deSUMOylation regulation of HIM but also suggest the potential of targeting this pathway to enhance cellular resilience under hypoxic stress.

## Introduction

Hypoxia, a state of reduced oxygen availability, is a main feature of ageing and is associated with age-related and ischaemic diseases. As the major consumers of oxygen for ATP production, mitochondria are particularly vulnerable to hypoxic stress. Impaired mitochondrial function under hypoxia leads to reactive oxygen species accumulation and organelle damage [1–3]. To maintain cellular homeostasis and ensure survival, cells mount a multifaceted response, including a selective removal of dysfunctional mitochondria through a process called hypoxia-induced mitophagy (HIM) [4]. However, the precise mechanisms regulating HIM remain largely elusive.

Post-translational modifications (PTMs) play crucial roles in fine-tuning protein function and cellular responses. Among these, SUMOylation, the reversible attachment of small ubiquitin-like modifier (SUMO) proteins to target lysines, stands out for its diverse regulatory effects [5, 6]. While five SUMO family members seem to exist in humans, SUMO1-3 are the validated conjugatable paralogues [7]. SUMO1 is ~45% identical to SUMO2 and SUMO3, which differ by only 3 amino acids therefore are often referred to as SUMO2/3 [7, 8]. Deconjugation (deSUMOylation) is mediated by dedicated SUMO proteases, including 6 Sentrin-specific proteases (SENP1-3 and SENP5-7) [9]. This dynamic SUMOylation/deSUMOylation cycle modulates protein activity, conformation, localisation, and interactions, impacting various cellular processes, including hypoxic adaptation [10].

The interplay between hypoxia and SUMOylation is evident but complex [11–13]. Hypoxia-inducible factor 1α (HIF-1α), a master regulator of mitochondrial adaptations under low oxygen, undergoes both SUMOylation and deSUMOylation by SENP1 and SENP3 [14–16]. Interestingly, SENP1-mediated deSUMOylation enhances HIF-1α stability and nuclear translocation, promoting target gene expression, including those encoding the HIM-mediating proteins BNIP3, Nix, and FUNDC1 [14, 15, 17–19]. This suggests a potential upstream role for SENP1 in regulating HIM pathway components. However, global SUMOylation levels and SENP expression remain largely unaltered under hypoxia, while deSUMOylation activity of both SENP1 and SENP3 appears inhibited [20, 21]. These observations highlight the intricate and context-dependent nature of hypoxia-induced SUMOylation regulation.

Interestingly, studies on iron chelation-induced Parkin-independent mitophagy, which occurs without HIF-1α activation [22], suggest alternative regulatory mechanisms for mitophagy. Our recent work has shown that SENP3 promotes this type of mitophagy by deSUMOylating FIS1, likely via facilitating FIS1 mitochondrial targeting [23]. However, the precise molecular mechanisms underlying this process remain largely unclear. While FIS1 is well established for its role in mitochondrial fission [24–26], its involvement in mitophagy is increasingly recognised [23, 27–29]. Notably, in Parkin-dependent mitophagy, the FIS1-TBC1D15/17 complex functions to limit excessive autophagosome recruitment [30, 31]. However, the roles of SENP3, FIS1, TBC1D15/17, and their interactions in the context of HIM in Parkin-lacking cells (such as HeLa cells) have not been explored previously.

Here, we investigated the roles of SUMOylation/deSUMOylation and SUMO proteases in mitophagy and cell survival in HeLa cells under hypoxic conditions. We demonstrate that global deSUMOylation, particularly deSUMO2/3-ylation, promotes basal mitophagy and HIM. The SUMO2/3-specific protease SENP3 promotes HIM through deSUMOylating FIS1. Conversely, SUMOylatable FIS1 is essential for hypoxia-induced FIS1-TBC1D17 complex formation, which inhibits HIM. Furthermore, depletion of SENP3 or FIS1 promotes hypoxia-induced cell death, and expressing a SUMO2/3-ylation-deficient FIS1 mutant abolishes SENP3 depletion-induced cell death under hypoxia. These findings suggest a novel mechanistic model where the SENP3-FIS1 axis promotes mitophagy, fine-tuned by TBC1D17, and contributes to cell survival under hypoxia.

## Results

### Hypoxia induces mitophagy and mild global deSUMO2/3-ylation

**Fig. 1:**
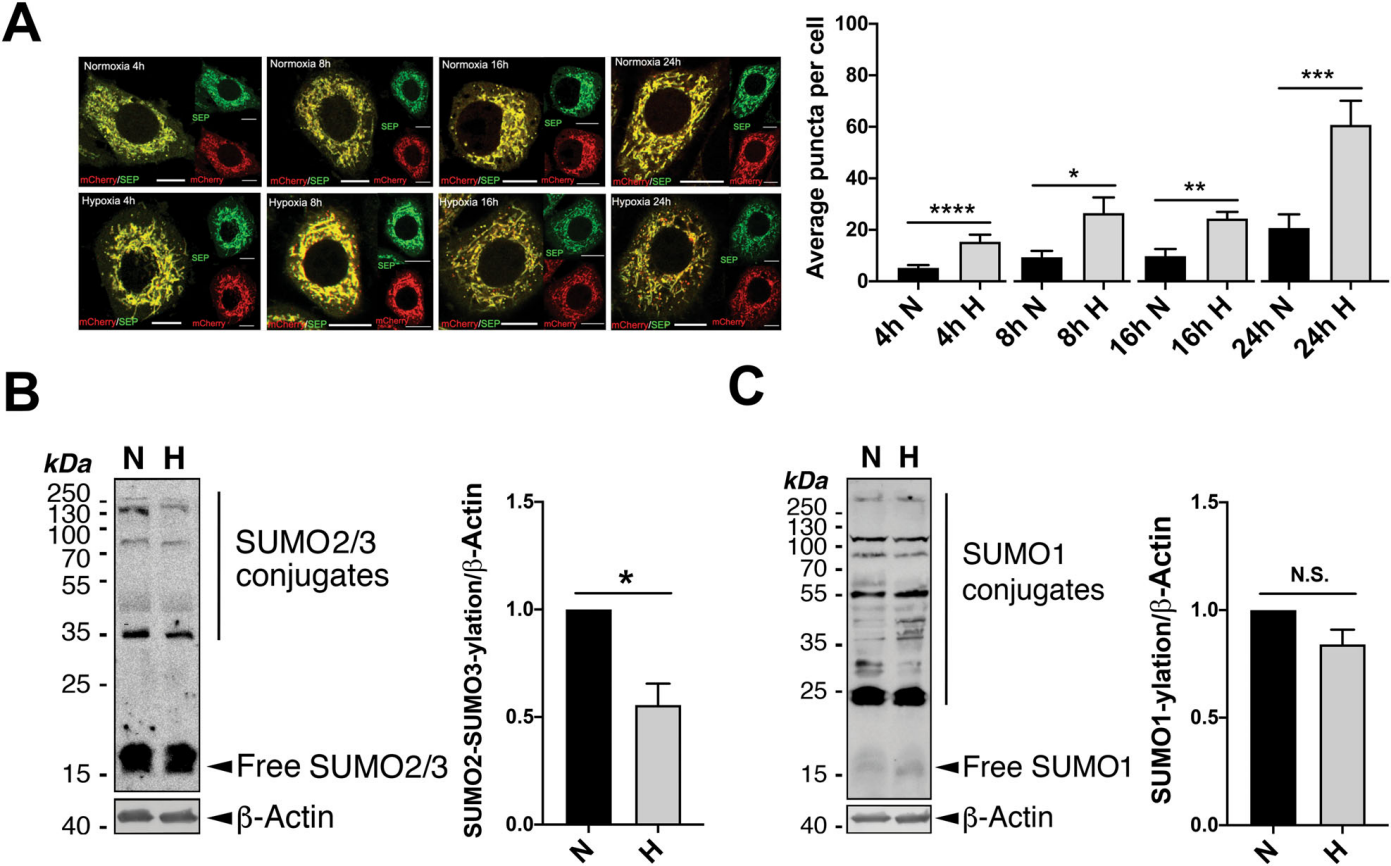
deSUMO2/3-ylation induces mitophagy and promotes HIM. **A** Hypoxia induces mitophagy. HeLa cells were transfected with Mito-pHfluorin per 35mm-dish and exposed to normoxia (N) or hypoxia (H;1% O_2_ for 4, 8, 16, or 24 h) (Scale bar, 10 μm). Upper panel shows that hypoxia-induced mitophagy is detectable as early as 4 h after cells exposed to 1% O_2_. Histogram in the right panel shows the average number of puncta per cell under indicated time points (*n* = 19 ~ 51 cells; *p < 0.05; **p < 0.01; ***p < 0.001; ****; unpaired t-test). **B**, **C** Hypoxia causes decreased SUMO2/3-ylation (**B**, *n* = 5, biological replicates; *p < 0.05; paired t-test) but not SUMO1-ylation (**C**, *n* = 6, biological replicates; N.S., non-significant; paired *t*-test) in HeLa cells. HeLa cells were exposed to 1% O_2_ for 24 h. Whole cell lysate samples were prepared and blotted as indicated.

**Fig. 2:**
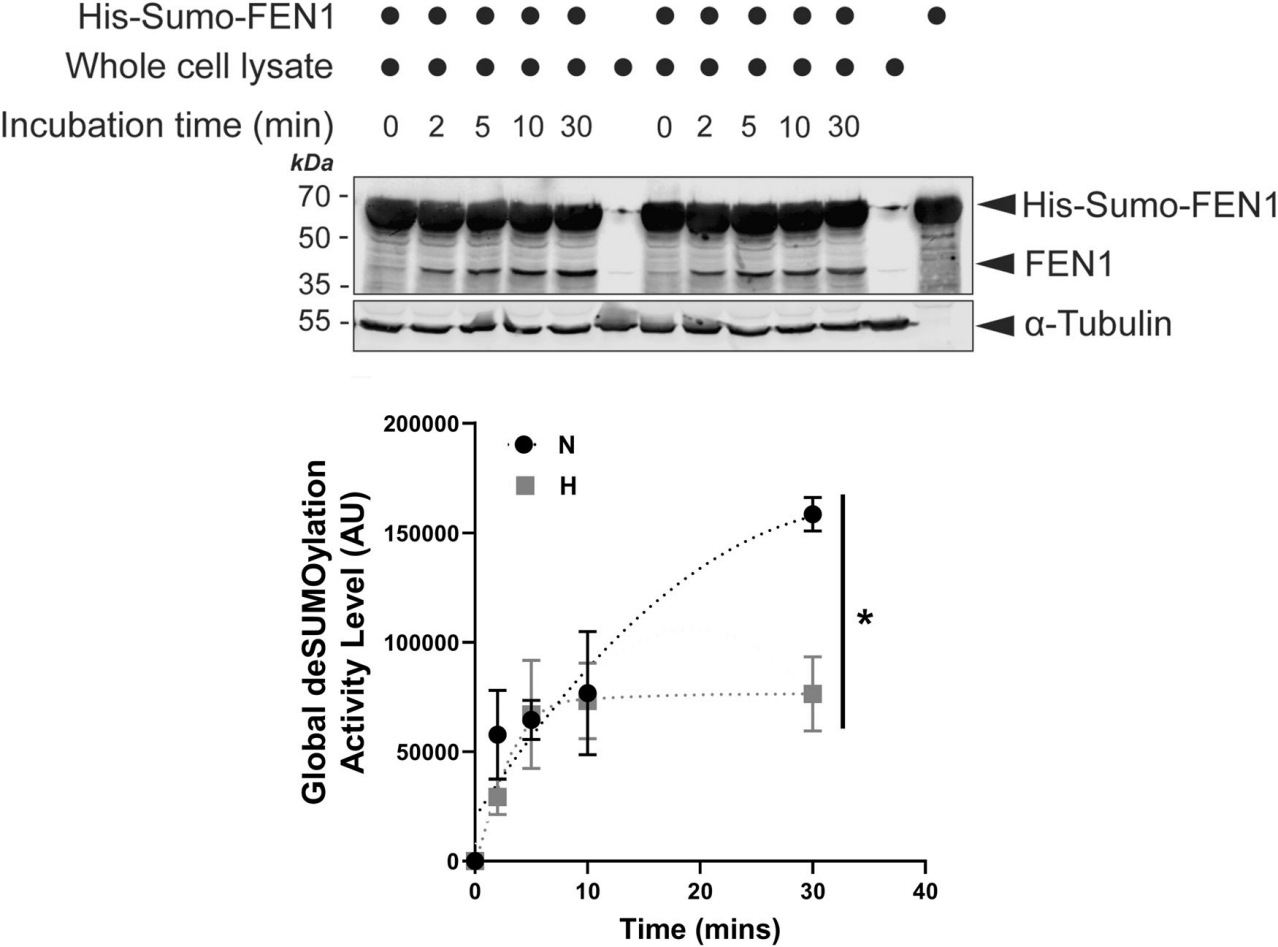
Hypoxia affects the global deSUMOylation activity levels. HeLa cells were cultured under either normoxic or hypoxic (1% O_2_) conditions for 24 h. Lysate samples from these cells were incubated with His-Sumo-FEN1 at 30 °C for varying durations. Lysate levels of FEN1, generated by SUMO protease cleavage of His-Sumo-FEN1, were detected by immunoblotting and normalised to α-Tubulin (loading control). The resulting FEN1 levels were expressed as an index of global deSUMOylation activity in arbitrary units (AU; *n* = 3, biological replicates; *p < 0.05; unpaired t-test).

Hypoxia is known to induce macroautophagy [32] and selective autophagy of intracellular organelles such as mitochondria [19]. Consistent with existing literature [19, 33, 34], we detected changes in the levels of autophagy markers LC3-II and p62 as well as mitochondrial marker Tom20 in model (HeLa) cells exposed to hypoxia (1% O_2_) for various durations (Fig. S1), suggesting the occurrence of macroautophagy and likely mitophagy. In order to minimise the observer’s subjectivity (bias) potentially involved in examining the effects of hypoxia on mitophagy induction, we developed a semi-automated computer program (Fig. S2; see Materials and Methods for details) for quantitative analysis of mitophagy levels (as indicated by the number of red puncta per cell) in fixed model cells expressing the validated dual-fluorescent probe Mito-pHfluorin [23]. In comparison to their normoxic counterparts (exposed to atmospheric levels of oxygen), cells exposed to 1% O_2_ for 4, 8, 16, or 24 h showed significantly increased mitophagy levels (Fig. 1A). Longer durations of exposure to hypoxia seem to lead to more noticeable red puncta in Mito-pHfluorin expressing cells. So, we chose 24 h as the optimal time point to examine HIM levels in the rest of the experiments described in this study. Moreover, interestingly, exposing cells to 1% O_2_ for 24 h also led to a mild but significant decrease in global SUMO2/3-ylation (Fig. 1B) but not SUMO1-lyation (Fig. 1C) in HeLa cells. We then reasoned that the observed decrease in SUMO2/3-ylation under hypoxic conditions might be attributed to either increased global deSUMOylation or decreased SUMO conjugation. To investigate this, we established a novel SUMO protease assay utilising a Sumo-Fen1 fusion protein as a substrate (Fig. S3). Surprisingly, our results suggest that overall cellular deSUMOylation activities are actually suppressed under hypoxic conditions (Fig. 2), aligning with a previous report of partially inhibited SENP1 and SENP3 activities in hypoxia [21]. Taken together, these findings indicate that HIM coincides with global deSUMO2/3-ylation, possibly due to decreased SUMO2/3 conjugation during hypoxia.

**Fig. 3:**
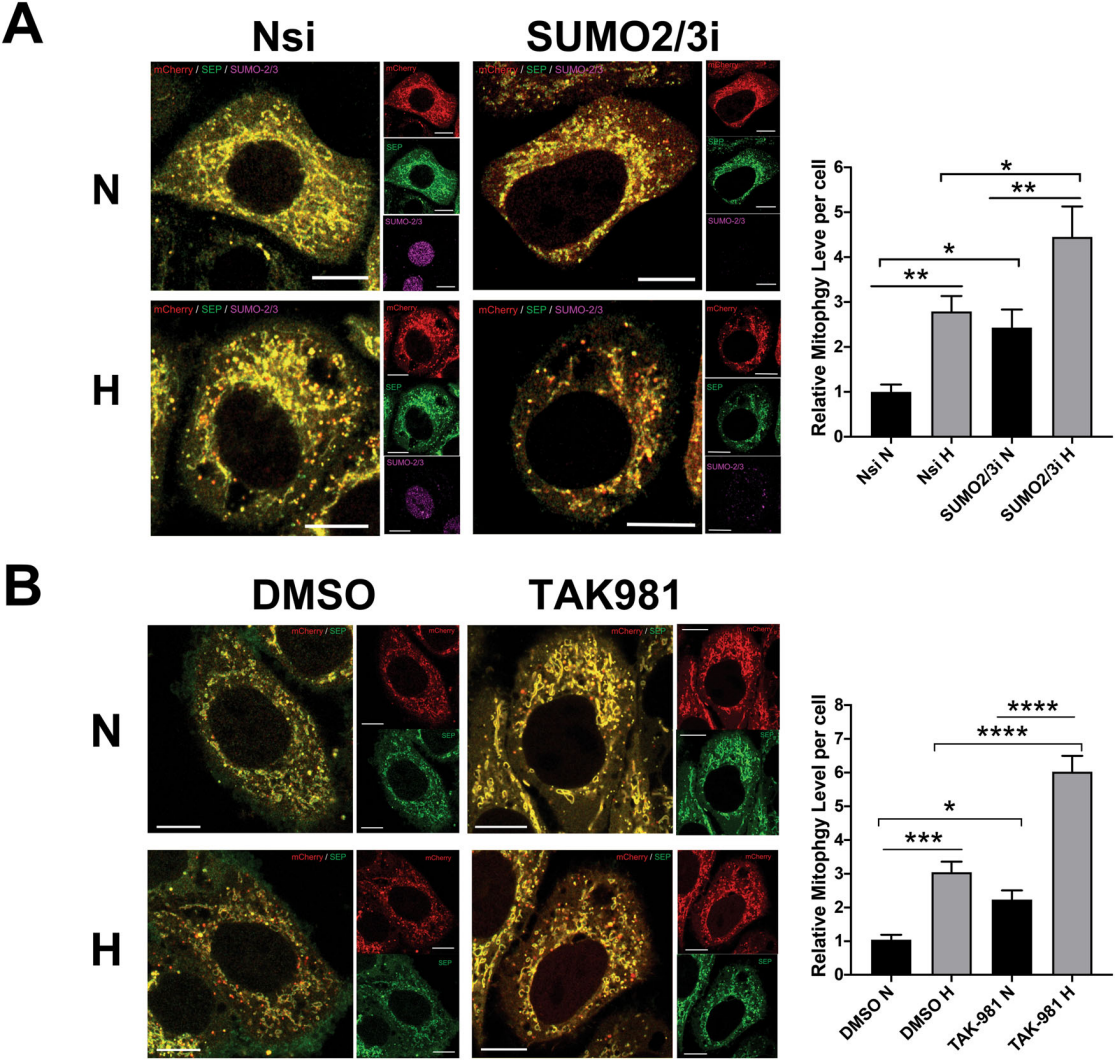
Global deSUMOylation induces mitophagy and promotes HIM. **A** RNAi-mediated SUMO-2/3 depletion induces mitophagy and promotes HIM. HeLa cells expressing Mito-pHfluorin were transfected with Nsi or SUMO-2/3-specific siRNA (SUMO-2/3i). 48 h post-transfection the cells were exposed to normoxia or hypoxia (1% O_2_) for 24 h, and the cells were analysed 72 h post-transfection (Scale bar 10 µm). Histogram in the right panel shows average number of Mito-pHfluorin red puncta per cell for cells exposed to N or H for 24 h (*n* = 42–71, *p < 0.05; **p < 0.01; Ordinary One-way ANOVA followed by Sidak’s multiple comparisons test). **B** Global SUMOylation inhibition induces mitophagy and promotes HIM. HeLa cells were transfected with Mito-pHfluorin. 48 h post-transfection the cells were treated with DMSO or TAK-981 (100 nM) and exposed to normoxia or hypoxia (1% O_2_) for 24 h, and the cells were analysed 72 h post-transfection (Scale bar 10 µm). Histogram in the right panel shows relative mitophagy level per cell for cells treated with DMSO or TAK-981 under and exposed to N or H for 24 h (*n* = 62–81, *p < 0.05; ***p < 0.001; ****p < 0.0001; Ordinary One-way ANOVA by Sidak’s multiple comparisons test).

To explore whether SUMO2/3 is involved in mitophagy regulation, we examined the effects of RNAi-mediated knockdown (KD) of SUMO2/3 on mitophagy in HeLa cells exposed to normoxia or hypoxia. Intriguingly, SUMO2/3 depletion induces mitophagy for cells under normoxic conditions and promotes HIM (Fig. 3A), raising the possibility of reduced global SUMOylation due to SUMO2/3 depletion to promote mitophagy. Thus, we investigated roles of SUMO conjugation as a whole in mitophagy regulation using a potent inhibitor for SUMOylation, TAK-981 [35]. Consistent with findings from other cell types in previous studies [36–38], treatment of cells with TAK-981 at various concentrations for 4 h effectively prevents conjugation of both SUMO1 and SUMO2/3 to target proteins in HeLa cells (Fig. S4A and S4B). Similar to the effects of RNAi-mediated SUMO2/3 depletion on mitophagy induction, TAK-981 treatment, which inhibits the levels of global SUMO1-ylation and SUMO2/3-ylation, significantly increases mitophagy levels under normoxic and hypoxic conditions (Fig. 3B). Altogether, these results suggest a novel role for global SUMOylation in general, especially SUMO2/3-ylation, in suppressing basal mitophagy as well as HIM in HeLa cells.

### DeSUMOylation enzyme SENP3 promotes HIM

**Fig. 4:**
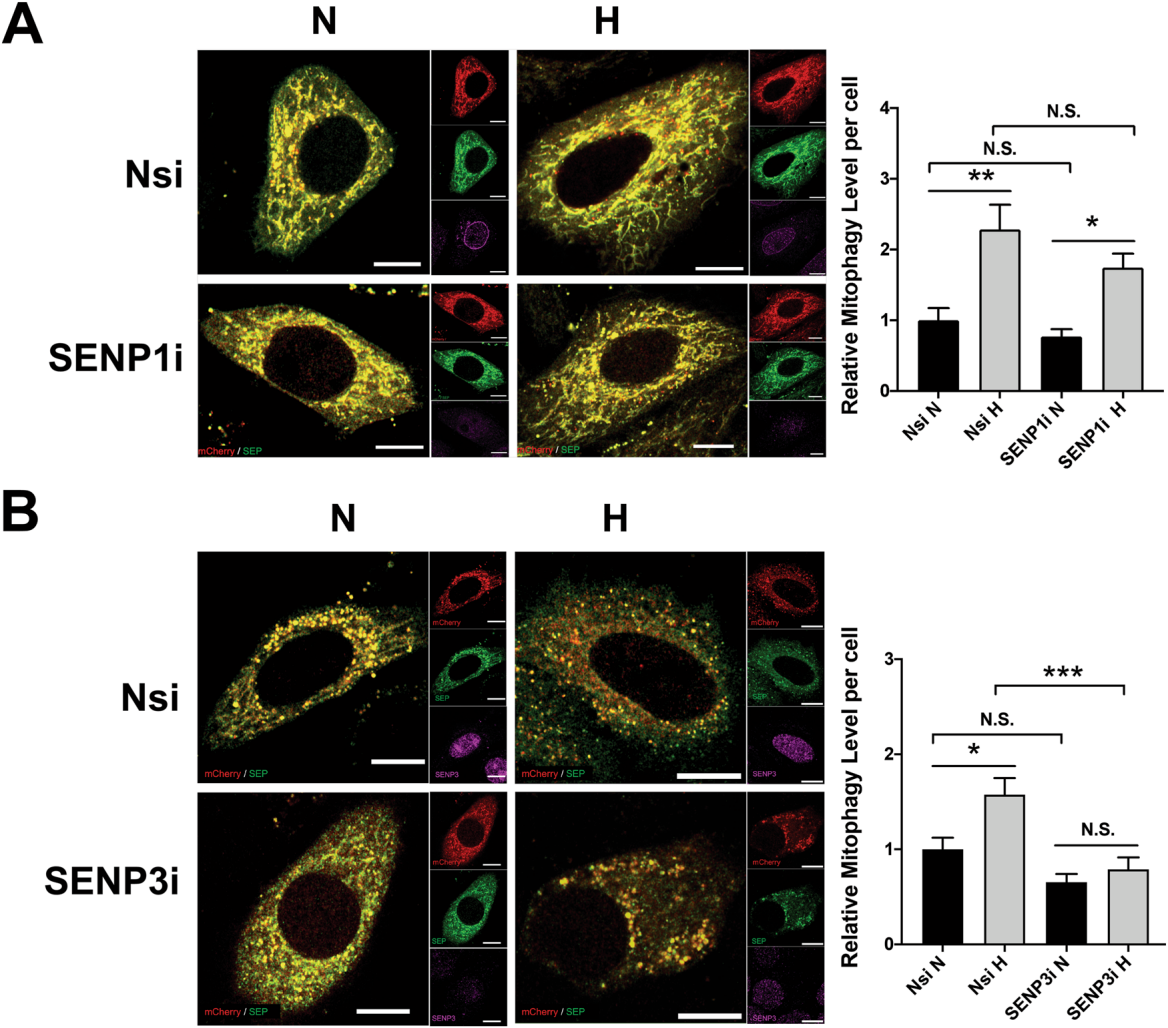
SENP3 plays an essential role in mitophagy induced by hypoxia. **A** RNAi-mediated SENP1 depletion does not appear to affect hypoxia-induced mitophagy in HeLa cells. HeLa cells expressing Mito-pHfluorin were transfected with Nsi or SENP1-specific siRNA (SENP1i). 48 h post-transfection the cells were exposed to normoxia or hypoxia (1% O_2_) for 24 h, and the cells were analysed 72 h post-transfection (Scale bar 10 µm). Histogram in the right panel shows relative mitophagy level per cell for cells exposed to N or H for 24 h (*n* = 32–55; N.S., non-significant; *p < 0.05; **p < 0.01; Ordinary One-way ANOVA by Sidak’s multiple comparisons test). **B** RNAi-mediated SENP3 depletion abolishes hypoxia-induced mitophagy in HeLa cells. HeLa cells expressing Mito-pHfluorin were transfected with Nsi or SENP3-specific siRNA (SENP3i). 48 h post-transfection the cells were exposed to normoxia or hypoxia (1% O_2_) for 24 h, and the cells were analysed 72 h post-transfection (Scale bar 10 µm). Histogram in the right panel shows average number of Mito-pHfluorin red puncta per cell for cells exposed to N or H for 24 h (*n* = 42–63, N.S., non-significant; *p < 0.05; ***p < 0.001; Ordinary One-way ANOVA by Sidak’s multiple comparisons test).

Previous studies have implicated at least two SUMO proteases, SENP1 and SENP3 in hypoxic cellular response [14, 21, 39], and that they are known as enzymes primarily responsible for deSUMO1-ylation and deSUMO2/3-ylation, respectively [9]. So, we examined if the two deSUMOlyation enzymes have roles in HIM regulation. Consistent with the findings from a previous report [21], exposing cells to 1% O_2_ for up to 24 h does not seem to lead to changes in SENP1 (Fig. S5A and S5B) and SENP3 levels (Fig. S6A and S6B). Interestingly, knockdown of SENP1 but not SENP3 appeared to reduce LC3-II induction in cells exposed to 1% O_2_ for 8 h (Fig. S3C and S4C), suggesting that SENP1 but not SENP3 may be necessary for LC3 lipidation, the phagophore expansion, and autophagosome formation upon any form(s) of autophagy induced by hypoxia. However, it should be noted that, since LC3 is not required in the initiation of autophagy but rather mediate autophagosome formation [40], the results does not rule out role(s) for SENP3 in the regulation of hypoxia-induced autophagy and we reasoned that SENP3 may be involved in other intracellular events essential for autophagy induction. To clarify roles for SENP1 and SENP3 in HIM, we examined mitochondria-containing autolysosome formation in control and SENP1-KD or SENP3-KD cells using mito-pHfluorin. Unexpectedly, SENP1 KD did not lead to significant changes in HIM levels (Fig. 4A), discounting the importance of SENP1 in this autophagic process. In contrast, SENP3 KD led to a significant decrease in HIM levels, indicating that SENP3 is essential for induction of mitophagy by hypoxia (Fig. 4B).

### FIS1 is essential for HIM

Since SENP3 is required for HIM, we next asked which protein(s) are potential downstream targets of SENP3-mediated deSUMOylation. The first protein that we investigated was FK506 binding protein 8 (FKBP8), a potential cytoplasmic target for SUMO2/3-ylation identified by mass spectrometry [41], and known to regulate HIM [42] as well as Parkin-independent mitophagy [43]. We validated FKBP8 as a bona fide target for SUMOylation (Fig. S7A) and found that exposure of cells to 24 h hypoxia decreases FKBP8 expression levels (Fig. S7B). However, unexpectedly, in contrast to the finding from a previous study [42], FKBP8 knockdown did not lead to a significant decrease in the levels of HIM (Fig. S7C), discounting the possibility that FKBP8 and SENP3 function in the same pathway.

**Fig. 5:**
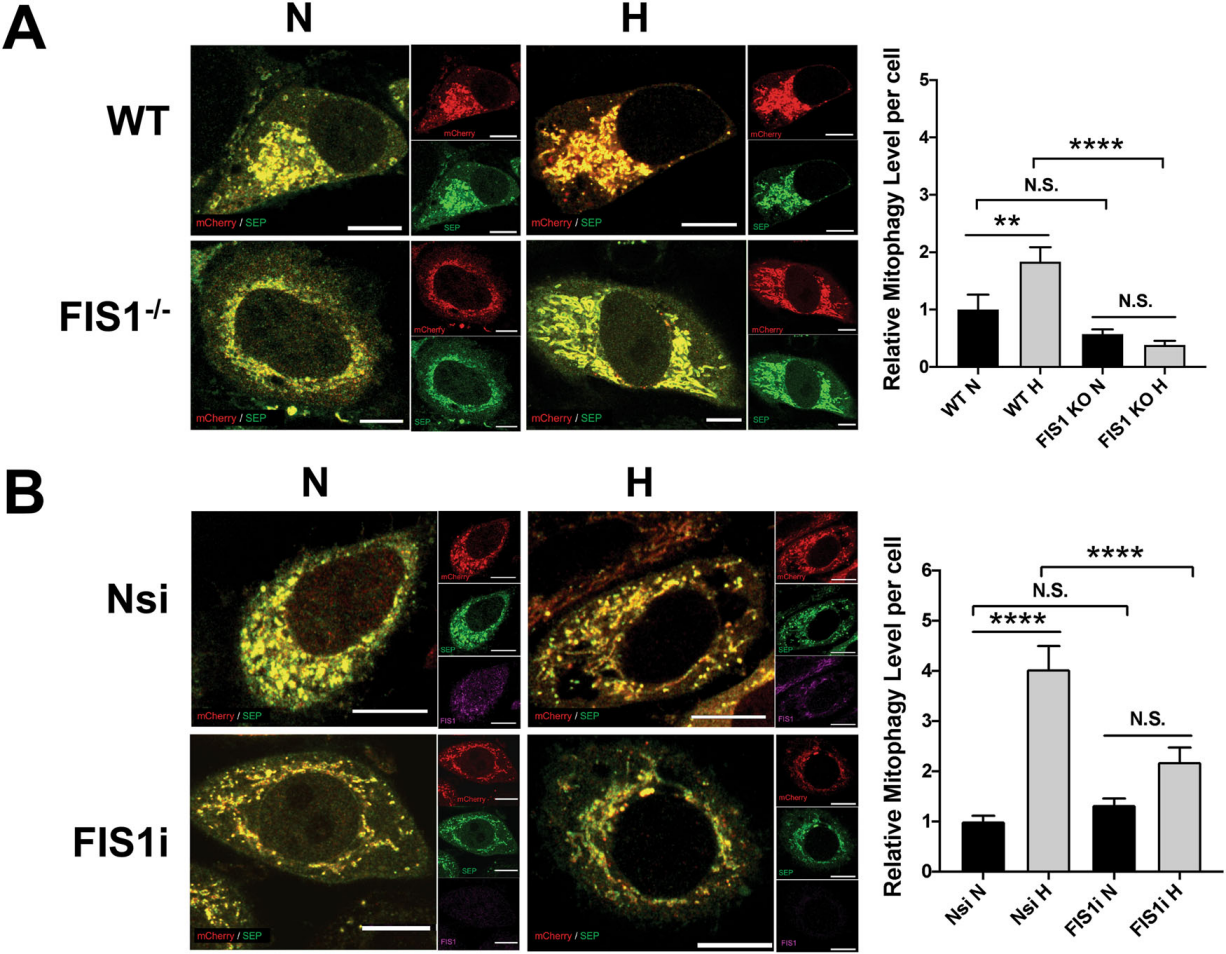
FIS1 is essential for mitophagy induced by hypoxia in HeLa cells. **A** Genetic depletion of FIS1 abolishes hypoxia-induced mitophagy. Wild-type (WT; FIS1^+/+^) or FIS1 knockout (KO; FIS1^−/−^) HeLa cells were transfected with Mito-pHfluorin. 48 h post-transfection the cells were exposed to normoxia or hypoxia (1% O_2_) for 24 h, and the cells were analysed 72 h post-transfection (Scale bar 10 µm). Histogram in the right panel shows relative mitophagy level per cell for cells exposed to N or H for 24 h (*n* = 33–64, N.S., non-significant; **p < 0.01; ****p < 0.0001; Ordinary one-way ANOVA followed by Sidak’s multiple comparisons test). **B** RNAi-mediated FIS1 depletion prevents hypoxia-induced mitophagy. HeLa cells were transfected with Mito-pHfluorin and Nsi or FIS1-specific siRNA, and the cells were analysed 72 h post-transfection (Scale bar 10 µm). Histogram in the right panel shows relative mitophagy level per cell for cells exposed to N or H for 24 h (*n* = 54–61, N.S., non-significant; ****p < 0.0001; Ordinary one-way ANOVA followed by Sidak’s multiple comparisons test).

The second protein that we investigated is FIS1, based on our previous work in which FIS1 was validated as a target for SUMOylation and SENP3-mediated deSUMOylation of FIS1 promotes deferiprone (DFP)-induced mitophagy [23]. Similar to the effect of SENP3 depletion on HIM, genetic depletion of FIS1 in HeLa cells abolished HIM (Fig. 5A), indicating that FIS1 is necessary for HIM induction. Moreover, RNAi-mediated depletion of FIS1 in HeLa cells significantly reduced HIM levels (Fig. 5B), further reinforcing the necessity of the protein in HIM. Altogether, these results indicate the possibility that FIS1 and SENP3 function in the same pathway.

### SENP3-FIS1 axis regulates HIM

**Fig. 6:**
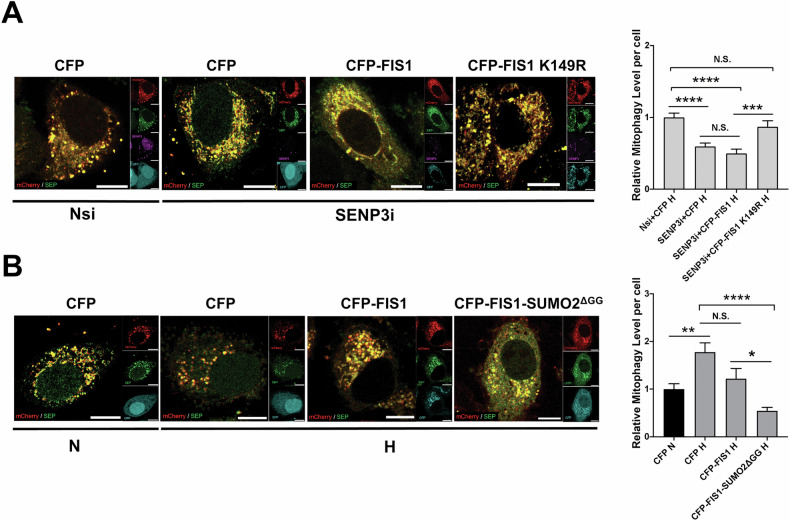
SUMOylatable FIS1 is required for SENP3 regulation of mitophagy induced by hypoxia. **A** Expressing SUMOylation-deficient CFP-FIS1 K149R rescues hypoxia-induced mitophagy in SENP3-KD HeLa cells. HeLa cells expressing Mito-pHfluorin were transfected with Nsi or SENP3i (50 nM), together with CFP, CFP-FIS1 or CFP-FIS1 K149R. 48 h post-transfection the cells were exposed to hypoxia (1% O_2_) for 24 h, and the cells were analysed 72 h post-transfection (Scale bar 10 µm). Histogram in the right panel shows relative mitophagy level per cell for cells exposed to N or H for 24 h (*n* = 48-74; N.S., non-significant; ***p < 0.001 ****p < 0.0001; Ordinary one-way ANOVA followed by Sidak’s multiple comparisons test). **B** Expressing or CFP-FIS1-SUMO2^ΔGG^ abolishes hypoxia-induced mitophagy. HeLa cells expressing Mito-pHfluorin were transfected CFP, CFP-FIS1 or CFP-FIS1-SUMO2^ΔGG^. 48 h post-transfection the cells were exposed to normoxia or hypoxia (1% O_2_) for 24 h. and the cells were analysed 72 h post-transfection (Scale bar 10 µm). Histogram in the right panel shows relative mitophagy level per cell for cells exposed to N or H for 24 h (*n* = 46–58; N.S., non-significant; *p < 0.05 **p < 0.01 ****p < 0.0001; Ordinary one-way ANOVA followed by Sidak’s multiple comparisons test).

We have previously demonstrated that SUMO2/3-ylation of FIS1 at K149 is required for SENP3 regulation of mitophagy induction by DFP [23], so we investigated if FIS1 SUMOylation status was also important for HIM regulated by SENP3. CFP, CFP-FIS1 WT or CFP-FIS1 K149R was expressed in SENP3-KD HeLa cells expressing mito-pHfluorin. As expected, mitophagy levels in cells expressing CFP-FIS1 K149R mutant were significantly higher than those in cells expressing CFP or CFP-FIS1 WT and were comparable to those in CFP expressing knockdown-control cells (Fig. 6A), indicating an essential rescue effect of the FIS1 SUMO2/3-ylation deficient mutant on the reduced mitophagic autolysosome formation due to SENP3 depletion under hypoxia and that SENP3 regulation of HIM is dependent on SUMOylatable FIS1. Conversely, we monitored HIM levels in HeLa cells expressing CFP, CFP-FIS1 or CFP-FIS1-SUMO2^ΔGG^ (a mutant mimicking constitutively SUMOylated form of FIS1). Expressing CFP-FIS1-SUMO2^ΔGG^ but not CFP or CFP-FIS1 significantly reduced HIM levels. Additionally, there was no significant difference in levels of mitophagy between cells expressing CFP-FIS1-SUMO2^ΔGG^ under hypoxia and the control cells expressing CFP under normoxia (Fig. 6B), implying that fully SUMOylated FIS1 has a suppressive effect on HIM. Altogether, these results indicate that SENP3-FIS1 axis regulates HIM likely through dynamically changing SUMOylation status of FIS1.

### FIS1 SUMO2/3-ylation is required for TBC1D17 regulation of HIM

**Fig. 7:**
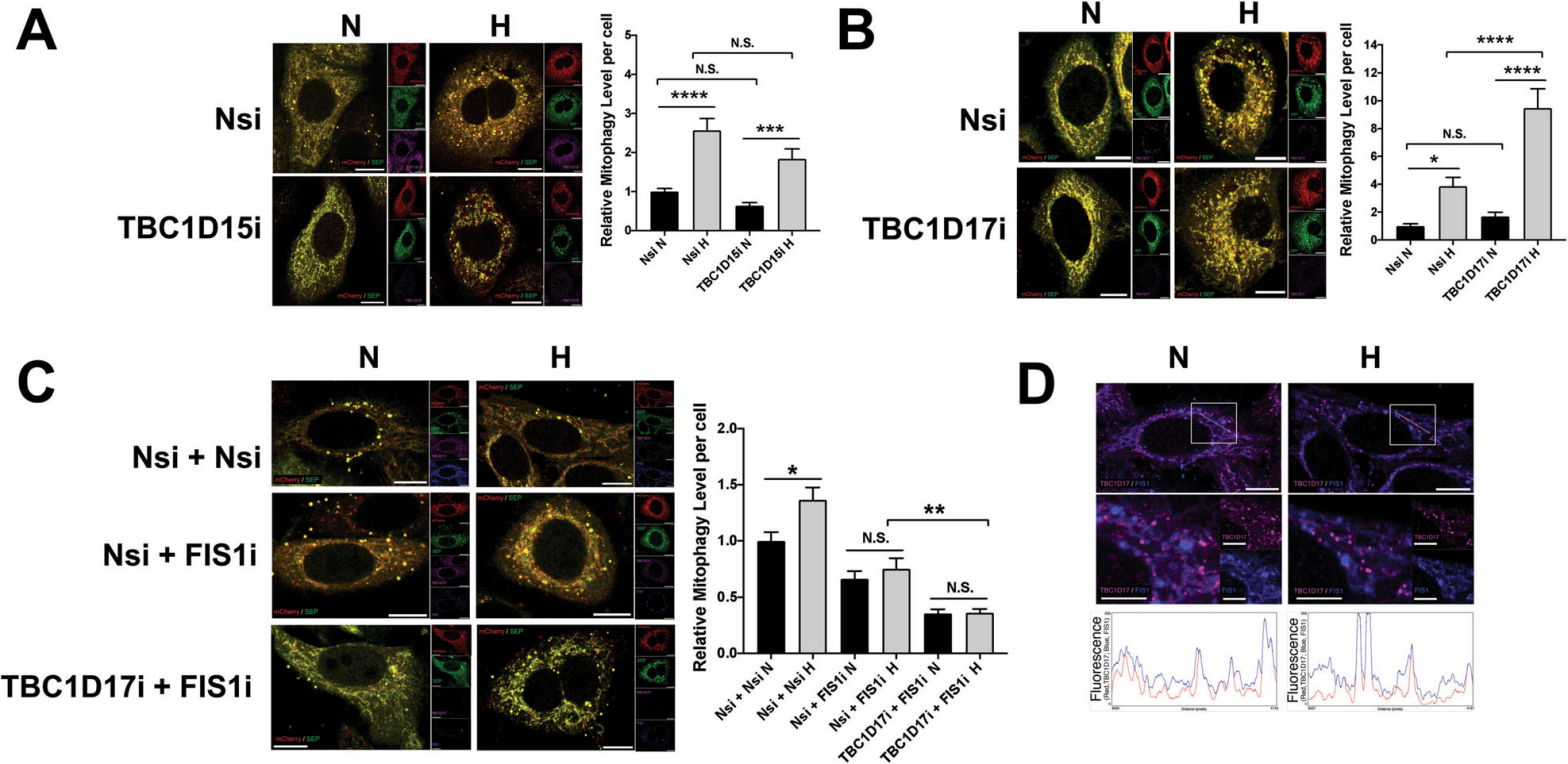
FIS1 is required for TBC1D17 regulation of HIM. **A** RNAi-mediated TBC1D15 depletion does not affect hypoxia-induced mitophagy in HeLa cells. HeLa cells were transfected with Mito-pHfluorin and Nsi or TBC1D15-specific siRNA (TBC1D15i; 20 nM). 48 h post-transfection the cells were exposed to normoxia or hypoxia (1% O_2_) for 24 h, and the cells were analysed 72 h post-transfection (Scale bar 10 µm). Histogram in the right panel shows relative mitophagy level per cell for cells exposed to N or H for 24 h (*n* = 39–59, N.S., non-significant; ***p < 0.001; ****p < 0.0001; Ordinary one-way ANOVA followed by Sidak’s multiple comparisons test). **B** RNAi-mediated TBC1D17 depletion promotes hypoxia-induced mitophagy in HeLa cells. HeLa cells were transfected with Mito-pHfluorin and Nsi or TBC1D17-specific siRNA (TBC1D17i; 20 nM). 48 h post-transfection the cells were exposed to normoxia or hypoxia (1% O_2_) for 24 h, and the cells were analysed 72 h post-transfection (Scale bar 10 µm). Histogram in the right panel shows relative mitophagy level per cell for cells exposed to N or H for 24 h (*n* = 42–56, N.S. non-significant; *p < 0.05; ****p < 0.0001; Ordinary one-way ANOVA followed by Sidak’s multiple comparisons test). **C** HeLa cells expressing Mito-pHfluorin were transfected with Nsi, TBC1D17i (20 nM), and/or FIS1-specific siRNA (50 nM). 48 h post-transfection the cells were exposed to normoxia or hypoxia (1% O_2_) for 24 h, and the cells were analysed 72 h post-transfection (Scale bar 10 µm). Histogram in the right panel shows relative mitophagy level per cell for cells exposed to N or H for 24 h (*n* = 52–62; N.S., non-significant; *p < 0.05; **p < 0.01; Ordinary one-way ANOVA followed by Sidak’s multiple comparisons test). **D** Hypoxia does not appear to affect the colocalisation of TBC1D17 with FIS1 in the Nsi/Nsi cells shown in (**C**). Relative fluorescence intensity of each channel as points along the white lines shown in the lower graphs for normoxia and hypoxia, respectively (Scale bar 10 µm).

**Fig. 8:**
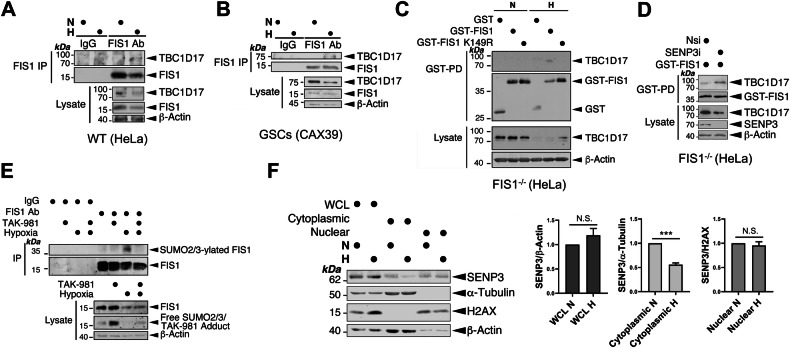
SUMOylatable FIS1 is required for FIS1-TBC1D17 interaction. **A**, **B** Hypoxia induces FIS1-TBC1D17 interaction. HeLa cells (A) or GSCs (B) were exposed to normoxia or hypoxia (1% O_2_) for 24 h. FIS1 was enriched through immunoprecipitation (IP). Lysate (input) and IP samples were immunoblotted as indicated. **C** TBC1D17 interacts with GST-FIS1 but not GST-FIS1 K149R mutant in FIS1 KO HeLa cells under hypoxia. FIS1 KO HeLa cells were transfected with GST, GST-FIS1 or GST-FIS1 K149R mutant. 48 h post-transfection the cells were exposed to normoxia or hypoxia (1% O_2_) for 24 h. GST-tagged proteins were enriched through GST-Pulldown (PD), Lysate (input) and GST-PD samples were immunoblotted as indicated. **D** SENP3 knockdown increases TBC1D17 interaction with GST-FIS1 in FIS1 KO HeLa cells exposed to hypoxia (1% O_2_) for 24 h. FIS1 KO HeLa cells expressing GST-FIS1 were transfected with Nsi or SENP3i. 48 h post-transfection the cells were lysed and GST-tagged proteins were enriched through GST-PD, Lysate (input) and GST-PD samples were immunoblotted as indicated. **E** Hypoxia induces FIS1 SUMO2/3-ylation in HeLa cells. HeLa cells were exposed to normoxia or hypoxia (1% O_2_) in the absence or presence of TAK981 (100 nM) for 24 h. FIS1 was enriched through IP. Lysate (input) and IP samples were immunoblotted as indicated. **F** Hypoxia reduces the levels of cytoplasmic SENP3 in HeLa cells. HeLa cells were exposed to normoxia or hypoxia (1% O_2_) for 24 h. Samples of whole cell lysate (WCL), cytoplasmic or nuclear fraction was prepared and blotted as indicated, SENP3 levels were normalised to β-Actin in WCL, α-Tubulin in the cytoplasmic fraction, and H2AX in the nuclear fraction (*n* = 5 biological replicates; N.S., not statistically significant; ***p < 0.001; Paired *t*-test).

Previous studies have identified exogenously expressed TBC1D15 and TBC1D17, two Rab GTPase-activating proteins (Rab-GAPs) that form hetero-dimers with each other, as the interactors for FIS1 [30, 31]. Exogenously expressed TBC1D15 and TBC1D17 have been shown to interact with FIS1 to limit autophagosome formation via inhibiting Rab7 activity in Parkin-dependent mitophagy [31, 44]. Therefore, we investigated whether FIS1 mediates HIM through its interaction with one or both two Rab-GAPs. RNAi-mediated depletion of TBC1D15 in HeLa cells led to no significant change in either HIM or mitophagy under normoxic conditions (Fig. 7A). On the other hand, RNAi-mediated depletion of TBC1D17 in HeLa cells significantly increased levels of HIM (Fig. 7B). These results do not support a role for TBC1D15 in regulating HIM and indicate that TBC1D17 suppresses HIM. Next, we further investigated whether TBC1D17 works with FIS1 to mediate this regulation. Thus, we performed a double KD of FIS1 and TBC1D17 in HeLa cells exposed to hypoxia for 24 h. Interestingly in FIS1 KD cells the depletion of TBC1D17 does not increase levels of HIM (Fig. 7C), indicating that suppression of HIM by TBC1D17 is dependent on FIS1. Since our imaging analysis showed that co-localisation of TBC1D17 with FIS1 did not seem to increase under hypoxia (Fig. 7D and S8), we investigated whether hypoxia had a role in regulating the interaction between the two proteins. Consistent with the observations made in previous studies [30, 31], endogenous FIS1-TBC1D17 interaction in HeLa cells under normoxic conditions appeared to be undetectable (Fig. 8A). Nevertheless, importantly, TBC1D17 was associated with FIS1 in HeLa cells at endogenous levels under hypoxic conditions (Fig. 8A), although they were downregulated in HeLa cells exposed to hypoxia for 24 h (Fig. S9). Importantly, the presence of hypoxia-inducible FIS1-TBC1D17 association was detected in GSC cultures derived from glioblastoma patients, established in vitro disease models [45–47] (Fig. 8B and S10), suggesting the relevance of this protein-protein interaction in the survival and maintenance of primary cancerous cells under hypoxia (a significant characteristic of the tumour microenvironment in many solid cancers). We then examined the mechanism underlying the association between FIS1 and TBC1D17. As expected, exogenously expressed GST-FIS1 in FIS1 KO HeLa cells was associated with endogenous TBC1D17 under hypoxic but not normoxic conditions (Fig. 8C), further substantiating that hypoxia induces FIS1-TBC1D17 interaction. Intriguingly, SUMOylation deficient mutant GST-FIS1 K149R expressed in FIS1 KO HeLa cells was not associated with endogenous TBC1D17 under hypoxic conditions (Fig. 8C), indicating that deSUMO2/3-ylation of FIS1 negatively regulates its interaction with TBC1D17. Conversely, SENP3 knockdown, known to enhance SUMOylation status of FIS1 [23], led to increased association of GST-FIS1 with endogenous TBC1D17 (Fig. 8D), suggesting that enhanced SUMO2/3-ylation of FIS1 positively regulates its interaction with TBC1D17. Indeed, exposure of HeLa cells to hypoxia led to an increase in FIS1 SUMO2/3-ylation (Fig. 8E). This finding is consistent with previous reports of decreased SENP3 levels in ischemic rat ventricular tissue and oxygen-glucose-deprived H9C2 cardiomyocytes [48]. Our biochemical analysis confirmed a significant reduction in SENP3 levels in the cytoplasmic fraction of HeLa cells under hypoxic conditions (Fig. 8F). Given that FIS1 is a known substrate for SENP3-mediated deSUMO2/3-ylation in the cytoplasm [23], these results suggest that the reduced availability of SENP3 may contribute to the hypoxia-induced increase in FIS1 SUMO2/3-ylation. Additionally, the observed global decrease in cellular deSUMOylation activities (Fig. 2), could further contribute to the accumulation of SUMO2/3 modifications on FIS1. Collectively, these findings indicate that increased FIS1 SUMO2/3-ylation, likely resulting from reduced SENP3 availability/activity, is a critical factor in the hypoxia-induced interaction between FIS1 and TBC1D17.

### SENP3-FIS1 axis protects cells against hypoxia-induced cell death

**Fig. 9:**
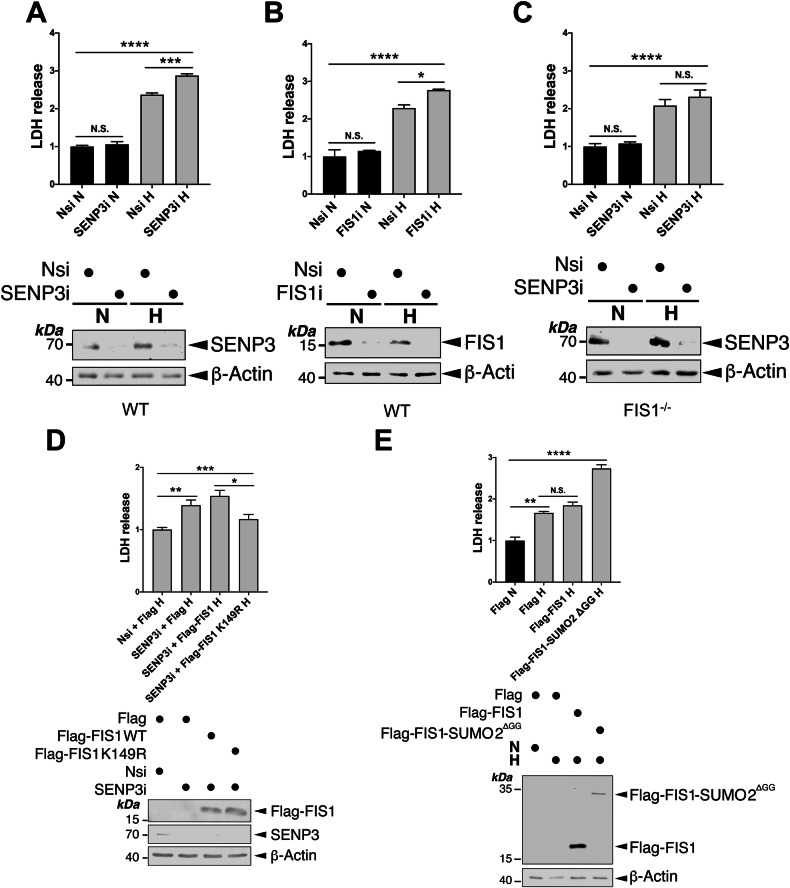
SENP3-FIS1 axis promotes cell survival under hypoxia. **A** RNAi-mediated depletion of SENP3 increases LDH release from HeLa cells exposed to hypoxia for 24 h. HeLa cells were transfected with Nsi or SENP3i. 48 h post-transfection the cells were exposed to normoxia or hypoxia (1% O_2_) for 24 h. Histogram in the upper panel shows relative levels of LDH release under indicated conditions (*n* = 3; biological replicates; N.S., non-significant; ***p < 0.001; ****p < 0.0001; Ordinary one-way ANOVA followed by Tukey’s multiple comparisons test). **B** RNAi-mediated depletion of FIS1 increases LDH release from HeLa cells exposed to hypoxia for 24 h. HeLa cells were transfected with Nsi or FIS1i. 48 h post-transfection the cells were exposed to normoxia or hypoxia (1% O_2_) for 24 h. Histogram in the upper panel shows relative levels of LDH release under indicated conditions (*n* = 3 biological replicates; N.S., non-significant; *p < 0.05; ****p < 0.0001; Ordinary one-way ANOVA followed by Tukey’s multiple comparisons test). **C** RNAi-mediated depletion of SENP3 does not increase LDH release from FIS1 KO HeLa cells exposed to hypoxia for 24 h. FIS1 KO HeLa cells were transfected with Nsi or SENP3i. 48 h post-transfection the cells were exposed to normoxia or hypoxia (1% O_2_) for 24 h. Histogram in the upper panel shows relative levels of LDH release under indicated conditions (*n* = 6 biological replicates; N.S., non-significant; ****p < 0.0001; Ordinary one-way ANOVA followed by Tukey’s multiple comparisons test). **D** Expressing SUMOylation-deficient FIS1 K149R mutant reverses the effect of SENP3 knockdown on hypoxia-induced LDH release. *pcDNA3*-Flag, Flag-FIS1 WT or Flag-FIS1 K149R mutant was transfected into HeLa cells in which SENP3 was depleted using siRNA for 48 h, and the cells were exposed to hypoxia (1% O_2_) for a further 24 h. Histogram in the upper panel shows relative levels of LDH release under indicated conditions (*n* = 5 ~ 6 biological replicates; *p < 0.05; **p < 0.01; ***p < 0.001; Ordinary one-way ANOVA followed by Tukey’s multiple comparisons test). **E** Expression of Flag-FIS1-SUMO2^ΔGG^, but not Flag-FIS1, in HeLa cells increases hypoxia-induced LDH release. *pcDNA3*-Flag, Flag-FIS1 WT or Flag-FIS1-SUMO2^ΔGG^ mutant was transfected into HeLa cells for 48 h, and the cells were exposed to normoxia or hypoxia (1% O_2_) for 24 h. Histogram in the upper panel shows relative levels of LDH release under indicated conditions (*n* = 3 biological replicates; N.S., non-significant; **p < 0.01; ****p < 0.0001; Ordinary one-way ANOVA followed by Tukey’s multiple comparisons test). In (**A**–**E**), whole cell lysate samples were immunoblotted as indicated in the lower panels.

Both SENP3 and FIS1 are known to be involved in cell survival pathways in different experimental settings [49–56]. In this study, our results indicate that the SENP3-FIS1 axis regulates HIM, which is regarded as a mitochondrial adaptation for cell survival [33, 57, 58]. Therefore, we investigate whether this axis has a role in cell survival under hypoxia using lactate dehydrogenase (LDH) release as a marker to monitor the levels of cell death. Either SENP3 KD or FIS1 KD led to a significant increase in LDH release from HeLa cells exposed to hypoxia for 24 h (Fig. 9A, B). Consistent with the role for SENP3 as the deSUMO2/3-ylating enzymes for FIS1 [23], SENP3 KD did not cause a significant increase in LDH release from FIS1 KO HeLa cells (Fig. 9C), supporting that FIS1 functions as a downstream target for SENP3-mediated cell death induced by hypoxia. Moreover, expressing Flag-FIS1 K149R mutant, but not Flag-FIS1, significantly reduced LDH release induced by SENP3 depletion in HeLa cells exposed to hypoxia for 24 h (Fig. 9D), indicating that maintaining FIS1 in deSUMOylation status favours cell survival under hypoxia. Conversely, expressing Flag-FIS1-SUMO2^ΔGG^, but not Flag-FIS1 in HeLa cells increases hypoxia-induced LDH release (Fig. 9E), suggesting that maintaining FIS1 in SUMOylation status promotes cell death under hypoxia. Altogether, these results indicate that the change of SUMOylation status of FIS1 is an important determinant of cell death and survival in HeLa cells under hypoxia.

## Discussion

**Fig. 10:**
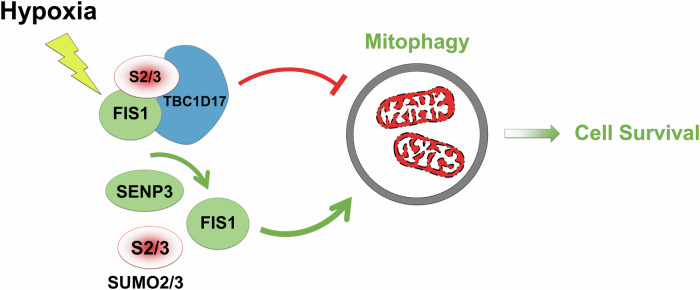
Schematic representation of a proposed SUMO2/3-dependent mitophagy/cell survival pathway under hypoxia. In response to hypoxia, FIS1 SUMO2/3-ylation increases due to reduced cytoplasmic SENP3. SUMO2/3-ylated FIS1 interacts with TBC1D17. With the associated FIS1, TBC1D17 inhibits the levels of hypoxia-induced mitophagy. Moreover, FIS1 deSUMO2/3-ylation mediated by residual cytoplasmic SENP3 is essential for maintaining hypoxia-induced mitophagy for cell survival.

A well-recognised phenomenon in cell biology, often referred to as the “SUMO enigma,” describes the observation that only a small fraction of any given protein is SUMOylated at any time. The balance between SUMOylating and deSUMOylating enzymes determines the extent of this modification [7]. As a result, proteins typically coexist in both SUMOylated and deSUMOylated//un-SUMOylated forms within cells. This raises the plausible hypothesis that these two forms might have distinct or even antagonistic functional consequences. Our findings support this hypothesis, revealing an antagonistic mechanism in cells under hypoxic conditions: (i) SUMOylation-dependent FIS1-TBC1D17 complex formation inhibits HIM, while (ii) deSUMOylation-dependent SENP3-FIS1 interaction promotes HIM. The hypoxia-triggered interaction between FIS1 and TBC1D17 may help maintain mitochondrial homeostasis by preventing excessive mitophagy, whereas the SENP3-FIS1 axis ensures sufficient removal of damaged mitochondria. Overall, SENP3, a protease known for its deSUMOylating activity on SUMO2/3-modified proteins, emerges as a key regulator in balancing SUMOylation and deSUMOylation of FIS1 to modulate HIM and promote cell survival (Fig. 10).

Our results demonstrate that hypoxia significantly reduces cytoplasmic SENP3 levels, correlating with increased SUMO2/3 modification of FIS1. However, contrary to a previous report of SENP3 translocation from the cytoplasm to the nucleus under ischemic stress [48], we did not observe a corresponding increase in nuclear SENP3 levels (Fig. 8F). Although SENP3 predominantly resides in the nucleus, it is possible that subtle changes in nuclear levels go undetected due to limitations in our subcellular fractionation and detection methods. Thus, this possibility cannot be entirely ruled out. Additionally, hypoxia may activate the PERK pathway, leading to lysosomal degradation of cytoplasmic SENP3 via cathepsins [53]. Our future research will focus on elucidating the mechanisms underlying the hypoxia-induced reduction of cytoplasmic SENP3.

To the best of our knowledge, this study is the first to report hypoxia-induced formation of the FIS1-TBC1D17 complex at endogenous levels. This interaction is dependent on SUMOylation of FIS1, as a SUMOylation-deficient FIS1 mutant failed to bind TBC1D17. Moreover, SENP3 depletion enhances FIS1-TBC1D17 complex formation, suggesting that SENP3 negatively regulates this interaction by deSUMOylating FIS1 under hypoxia. While the precise mechanism by which SUMO2/3 modification modulates FIS1 interaction with TBC1D17 remains unclear, two possibilities emerge. First, SUMOylation may promote FIS1 localisation to the ER [23], where it encounters TBC1D17, potentially recruited to the same compartment by hypoxia. Alternatively, SUMOylation may induce a conformational change in FIS1, exposing binding sites for TBC1D17.

Although TBC1D17 has been implicated in Parkin-dependent mitophagy [31], its precise role remains enigmatic. This study unveils a novel function for TBC1D17 in suppressing mitophagy in a FIS1-dependent manner. The mechanism by which the FIS1-TBC1D17 complex inhibits mitophagy remains speculative but it may involve diverting autolysosomes away from mitochondria via TBC1D17 interaction with SUMO2/3-ylated FIS1. Further investigation is needed to fully elucidate this pathway.

Hypoxia is a hallmark of the tumour microenvironment in solid cancers, including cervical cancer and glioblastoma multiforme (GBM), the most aggressive form of glioma [59–62]. Hypoxia triggers adaptive responses that enable cancer cells to survive under low-oxygen conditions, often resulting in increased tumour aggressiveness and therapy resistance. Our study provides evidence that the FIS1-TBC1D17 complex forms as part of the cellular response to hypoxia in tumour cells, specifically in clonal cervical cancer HeLa cells and GSCs. This complex may influence mitochondrial quality control, altering metabolism and promoting cancer cell survival. Interestingly, we did not observe this complex formation in primary cell cultures derived from renal cancer patients (Fig. S11), suggesting that the hypoxic response might be cell-type dependent, potentially due to heterogeneity in the tumour microenvironment. Further research is needed to explore these variations and their therapeutic implications.

Importantly, our findings also reveal that global deSUMOylation, rather than increased SUMOylation, is associated with mitophagy induction under hypoxia. Specifically, depletion of SUMO2/3 or inhibition of its conjugation with TAK-981 significantly enhances mitophagy, indicating that SUMOylation plays a suppressive role. Conversely, SENP3-mediated deSUMOylation of FIS1 appears essential for mitophagy induction under hypoxic conditions. While under hypoxia SENP3 is not stabilised, as seen with DFP-induced iron chelation [23], and its activities might be partially inhibited [21], residual SENP3 activity seems sufficient for FIS1 deSUMOylation to facilitate mitophagy. This suggests that SENP3 preferentially targets FIS1 for deSUMOylation when SUMO2/3-ylation levels are within a critical threshold. Importantly, our studies on Parkin-deficient (HeLa) cells reveal that the SENP3-FIS1 axis is required for mitophagy induced by both iron chelation and hypoxia. However, mechanistic differences between these two types of mitochondrial quality control processes are evident. Further in-depth investigation is needed to elucidate the underlying regulatory networks in each condition.

The role of SENP3 in cell survival versus death appears to be context-dependent, as previous studies have linked SENP3 to both processes depending on the conditions [48, 53, 54, 56, 63–67]. This study adds another layer to this complexity by revealing a protective role for SENP3-FIS1 axis in promoting cell survival in cells exposed to prolonged hypoxia (24 h). This finding may explain the seemingly contradictory observations regarding SENP3 roles. In brief, SENP3-FIS1 axis may promote cell survival by facilitating mitophagy under prolonged hypoxia [48, 67], while SENP3-DRP1 axis may promote cell death through mitochondrial fragmentation under shorter hypoxia durations plus glucose deprivation and followed by reoxygenation [53, 54, 56, 63–66]. Further research is required to fully understand how the SENP3-FIS1 axis promotes cell survival under hypoxia.

Last but not least, from a technological standpoint, this study introduced a novel assay to quantitatively measure the overall activity of SUMO proteases within cell lysates. Our results revealed a distinctive enzymatic kinetics profile for the cleavage of the C-terminal Gly-Gly motif of Sumo fused with FEN1 by active deSUMOylating enzymes, including SENPs, present in cell extracts (Fig. S3A). We observed an initial velocity phase during the first 10 min (2, 5, and 10 min time points), followed by a steady-state phase by 30 min. This kinetics pattern suggests that exposure of cells to hypoxia can decrease the maximum velocity (Vmax) of cellular deSUMOylating enzymes, including SENPs (Fig. 2 and S3B). The implications and applications of this assay extend beyond the scope of this study. It can be widely employed to assess global SUMO protease activity levels in various cellular and tissue samples, thereby contributing to fundamental and preclinical/clinical scientific research, as well as biomarker discovery and disease diagnosis.

In summary, we uncover previously unknown regulatory mechanisms involving global deSUMO2/3-ylation, SENP3-mediated FIS1 deSUMO2/3-ylation, and hypoxia-induced FIS1-TBC1D17 complex formation in controlling mitophagy levels. This intricate interplay between SUMOylated and un-/de-modified FIS1 allows cells to adapt to fluctuating oxygen levels and maintain optimal mitophagy for survival. Further unraveling this intricate network of protein interactions and regulatory mechanisms holds promise for understanding cellular adaptation to hypoxia, identifying potential therapeutic targets, and ultimately developing treatment strategies for hypoxia-related diseases.

## Materials and Methods

### Plasmids and mutagenesis

DNA constructs encoding mito-pHfluorin, GST-FIS1, Flag-FIS1, Flag-FIS1 K149R and Flag-SUMO2^ΔGG^ were described previously [23, 56]. Flag-tagged CFP, CFP-FIS1, CFP-FIS1 K149R and CFP-SUMO2^ΔGG^ were made by insertions of cDNA-encoding CFP into the BamHI/EcoRI sites of *pcDNA3*-Flag, Flag-FIS1, Flag-FIS1K149R and Flag-SUMO2^ΔGG^ [23], respectively. His-SUMO2 was provided by M. Roussel (Addgene Plasmid #133771) [68]. HA-FKBP8 was gifted by Y. K. Jung [42]. GST-FIS1 K149R mutant was made by PCR-based mutagenesis. His-tagged Sumo-FEN1 DNA construct, a gift from S.M. Hamdan [69], contains a double His6-tag, followed by the sequence encoding residues 3-98 of *S. cerevisiae* SMT3 (Sumo), and then the sequence encoding residues 2-379 of human FEN1.

### Cell culture

WT or FIS1 KO HeLa cells and HEK293 cells were maintained in Dulbecco’s modified Eagle’s medium (DMEM) (Lonza) containing 10% foetal bovine serum (FBS), 5 mM glutamine, and 100 units/ml penicillin/streptomycin at 37 °C in humidified ambient air supplemented with 5% CO_2_ (v/v) as described previously [23].

Primary GSC cultures (CX18 core 1, CX25 core 1 and CAX39 core 1 cell lines) were derived directly from surgically resected tumour tissues at the Royal Hallamshire Hospital (Ethical approval: Yorkshire & The Humber – Leeds East REC (11-YH-0319/STH15598)), as reported previously [45–47]. The GSCs were maintained in Dulbecco’s Modified Eagle Medium/Ham’s F-12-based ‘stem’ culture media containing 10% FBS, 1% B27, 0.5% N_2_, 1% L-glutamine, 1% Penicillin-streptomycin, 0.1% amphotericin B, 4 ug/mL heparin, 20 ng/mL epidermal growth factor and fibroblast growth factor at 37 °C in humidified ambient air supplemented with 5% CO_2_ (v/v), as described previously [45–47]. Cell cultures of passages 10 (of CX25 core 1), 14 (of CAX39 core 1) and 17 (of CX18 core 1) were used for experiments.

Primary renal cell cultures (EVD0061, EVD0062 and EVD0063) were derived directly from surgically resected tumour tissues from three patients at the Royal Hallamshire Hospital as part of the EVIDENT trial (REC: 20SW0193), and maintained in Roswell Park Memorial Institute (RPMI) 1640 medium, 10% FBS, and 5 mM glutamine, and 100 units/ml penicillin/streptomycin at 37 °C in humidified ambient air supplemented with 5% CO_2_ (v/v). Cell cultures of passages 3 and 4 were used for experiments.

### DNA and siRNA transfections

DNA, siRNA or DNA & siRNA were transfected into HeLa cells, FIS1 KO HeLa cells or HEK293 using jetPRIME (Polyplus Transfection). siRNA duplexes used were as follows: non-specific siRNA (Eurofins Genomics), FIS1 siRNA used previously [23], FKBP8 siRNA (duplexes to target GAGUGGCUGGACAUUCUGG to silence FKBP8; synthesised by Eurofins Genomics), SENP1 siRNA (Santa Cruz sc-44449), SENP3 siRNA (Santa Cruz, sc-44451), SUMO1 siRNA (duplexes to target CCUUCAUAUUACCCUCUCC to silence SUMO1; synthesised by Eurofins Genomics), SUMO2/3 siRNA (duplexes to target GUCAAUGAGGCAGAUCAGA to silence SUMO2/3 [70]; synthesised by Eurofins Genomics), TBC1D15 siRNA (duplexes to target UCAACAAGAAGAACCAGG to silence TBC1D15; synthesised by Eurofins Genomics) and TBC1D17 siRNA (Santa Cruz sc-97889).

### Hypoxia induction

Hypoxia treatment of the cells was performed within a SCI-tive hypoxia workstation (Baker Ruskinn) set to humidified 1% (v/v) O_2_, and 5% (v/v) CO_2_ at 37 °C. The DMEM medium mentioned above was degassed in the hypoxia workstation to remove the oxygen dissolved in the medium at least 24 h prior to placing cells into the hypoxia workstation. As the cells were placed into the workstation, the media in the sample dishes was replaced with the degassed DMEM medium. The cells were left in the hypoxia workstation for appropriate time durations. Control cells were cultured using a conventional mammalian cell culture incubator in normoxic conditions (*i.e*., normal ambient O_2_ tension: 20.9% (v/v)) for the same time durations. At the end of culture, cells were removed from the hypoxia workstation for either immunoblotting or immunofluorescence imaging analysis.

### Preparation of recombinant proteins, whole cell lysates, GST-PD, IP and nickel affinity purification

His6-Sumo-Fen 1 was produced from the vector pET His6-Sumo-Fen1 using BL21(DE3) *E. coli* as described previously [69]. Whole cell lysates were prepared from cells lysed with a buffer containing 20 mM Tris, pH 7.4, 137 mM NaCl, 25 mM β-glycerophosphate, 2 mM sodium pyrophosphate, 2 mM EDTA, 1% Triton X-100, 10% glycerol, and 1 × protease inhibitor cocktail (Roche) in the presence of 20 mM freshly made N-Ethylmaleimide (NEM) (Sigma). To enrich and isolate GST or GST-tagged proteins, GST-PD was conducted by incubating whole cell lysates with glutathione-sepharose 4B beads (Generon). To detect the interaction between FIS1-TBC1D17, IP was conducted by incubating whole cell lysates overnight with a rabbit polyclonal FIS1 antibody (Proteintech 10956-1-AP) pre-bound to protein-A beads (Sigma). To isolate His-SUMO2 conjugates, nickel affinity purification was conducted by incubating Ni-NTA agarose beads (Qiagen) with denatured lysate samples. Bead-bound proteins, obtained from the experimental procedures of GST-PD, IP, or nickel affinity purification, were subsequently eluted in SDS sample buffer for immunoblot analysis.

### Subcellular fractionation

Whole-Cell lysate, cytoplasmic or nuclear fraction samples from HeLa cells were prepared using a Nuclear Extraction kit (CHEMICON INTERNATIONAL).

### Immunoblotting

SDS-PAGE (7.5–15% gels) was run to separate protein samples that were then transferred to Immobilon-P membranes (Millipore Inc.) and incubated with primary antibodies. The primary antibodies used were: β-actin (mouse monoclonal Ab; Sigma-Aldrich A2228; 1:20,000 dilution; rabbit polyclonal Ab; ProteinTech 20536-1-AP; 1:5,000 dilution), FEN1 (mouse monoclonal Ab; Santa Cruz biotechnology sc-28355, 1:500 dilution), FIS1 (mouse monoclonal Ab; ProteinTech 66635-1-Ig; 1:1,000 dilution), GAPDH (mouse monoclonal Ab; Santa Cruz biotechnology sc-365062; 1:1,000 dilution), GST (goat polyclonal Ab; GE Healthcare; 1:10,000 dilution), HA-tag (rabbit polyclonal Ab; ProteinTech 66006-2-Ig, 1:2,000 dilution), HIF1α (mouse monoclonal Ab, BD Bioscience 610959; 1:1,000 dilution), His (rabbit polyclonal Ab, Cell Signaling #2365; 1:1,000 dilution), LC3 (rabbit polyclonal Ab, Cell Signaling #4108; 1:2,000 dilution), SENP1 (recombinant monoclonal Ab, Abcam EPR3844, 1:2,000 dilution), SENP3 (rabbit monoclonal Ab, Cell Signaling #5591, 1:10,000 dilution), SUMO1 (rabbit polyclonal Ab, Cell Signaling #4930; 1:1,000 dilution), SUMO2/3 (rabbit monoclonal Ab, Cell Signaling #4971; 1:1,000 dilution), TBC1D17 (rabbit polyclonal Ab, ProteinTech 20482-1-AP; 1:1,000 dilution), and α-Tubulin (rabbit polyclonal Ab, ProteinTech 11224-1-AP; 1:2,000 dilution or mouse monoclonal Ab, ProteinTech 66031-1-Ig; 1:20,000 dilution). Protein detection was performed either using fluorescent secondary antibodies (LI-COR) by a LI-COR imaging system or using HRP-conjugated secondary antibodies (Sigma) or an HRP-conjugated VeriBlot secondary antibody (Abcam ab131366 for immunoblotting of IP samples) followed by enhanced chemiluminescence (GE Healthcare Amersham). Every immunoblot (see full length blots in the ‘Supplemental Material’ file) shown represents the result of at least three separate experiments that were conducted with different cell populations. After quantification of fluorescently developed blots using LI-COR Image Studio, protein levels of interest were normalised to β-actin or GAPDH loading control levels. Each value is expressed as the mean ± SEM and expressed as a percentage of the control value.

### Fluorescence and microscopy imaging

HeLa cells seeded on 35 mm ibidi µ-dishes. After 24 h cells were exposed to hypoxia for indicated durations. For experiments involving cDNA/siRNA transfections, cells seeded on the dishes were transfected. 48 h post-transfection cells exposed to hypoxia for 24 h. At the end of culture, cells were fixed with 4% paraformaldehyde in PBS for 10 min at room temperature. Immunostaining was carried out as described previously [23]. The primary antibodies used were: FIS1 (rabbit polyclonal Ab; Proteintech 10956-1-AP; 1:100 dilution), FKBP8 (mouse monoclonal Ab; Santa Cruz Biotechnology sc-166607, 1:200 dilution), SENP1 (recombinant monoclonal Ab; Abcam EPR3844, 1:200 dilution), SENP3 (rabbit monoclonal Ab; Cell Signaling #5591, 1:500 dilution), SUMO1 (rabbit polyclonal Ab; Cell Signaling #4930, 1:250 dilution), SUMO2/3 (rabbit monoclonal Ab; Cell Signaling #4971, 1:100 dilution), TBC1D15 (rabbit polyclonal Ab; ProteinTech, 1:100 dilution), and TBC1D17 (rabbit polyclonal Ab; ProteinTech, 1:100 dilution). The secondary antibodies used were: Alexa Fluor 405 (A31553) and Alexa Fluor 680 (A32802) fluorescent secondary antibodies (ThermoFisher Scientific, 1:2,000). After incubation with the secondary antibody, the samples were washed thrice with 1× PBS. Fresh 1× PBS was added to the sample dishes. Samples were then kept at 4 °C covered by foil until microscopy. All immunofluorescence microscopy was performed with a Zeiss LSM 880 Airyscan confocal microscope within the Wolfson Light Microscope Facility at the University of Sheffield.

### Quantification of mitophagy probed by Mito-pHfluorin

To avoid potential human subjectivity in imaging analysis, a Macro program on ImageJ/Fiji automating the quantification of mitophagy as indicated by Mito-pHfluorin was generated. Procedures for quantification of the red puncta are outlined in Fig. S2 using the following computer programme:

if (isOpen(“Results”)){

close(“Results”);

}

MaxProm = getNumber(“Please enter threshold value for Maxima”, 1500);

//Ask user if they want to analyse another particle

while (getBoolean(“Do you wish to analyse a cell?”, “Yes”, “No, I’m done…”)==1){

//Set the freehand tool as active

setTool(“polygon”);

//Pause and wait for user to draw a selection

waitForUser(“Draw around a cell then click OK”);

//Exit macro if no selection made

if(selectionType() == −1){

exit(“No selection made”);

}

run(“Find Maxima…”, “prominence = “+MaxProm + “ output=Count”);

run(“Select None”);

}

The levels of mitophagy were analysed by either comparing the average red puncta per cell (for Fig. 1A) or comparing a normalised relative value per cell in relation to their control value per cell (for Figures other than Fig. 1A). Each value is presented as the mean ± SEM and expressed as a percentage of the control value.

### SUMO protease assay

A novel assay was developed to quantitatively measure the collective deSUMOylating activity (*i.e*., global SUMO protease activity) under various experimental conditions. The assay leverages the recognition of the tertiary structure of the C-terminal Gly-Gly motif of a SUMO moiety by active deSUMOylating enzymes, including SENPs, present in whole cell lysates. These enzymes cleave the peptide bond between the Gly residue and the following amino acid within a His6-tagged Sumo-FEN1 substrate. Ubiquitin-like-specific protease 1 (Ulp1; a kind gift from R. Ley and J. Grasby, Department of Chemistry, University of Sheffield), a *S. cerevisiae* SUMO protease and the evolutionary origin of SENP1, SENP2, SENP3, and SENP5 [71], served as a positive control.

Whole cell lysates were prepared in a lysis buffer containing 0.25 M sucrose, 20 mM MOPS-KOH (pH 7.4), 1 mM EDTA-NaOH pH 8, with 1 × protease inhibitor cocktail and DTT (1 mM), on ice with or without NEM (20 mM). After brief sonication, protein concentrations were determined using the Bradford assay. 1 μg of His-Sumo-FEN1 was added to 20 μl of relevant whole cell lysate (approximately 1 μg/μl) and incubated at 30 °C for varying durations. Reactions were terminated by adding sample buffer and analysed by SDS-PAGE under reducing and denatured conditions. Immunoblotting with a FEN1 antibody detected the levels of FEN1 without the His-tagged Sumo moiety, indicating the activity of deSUMOylating enzymes in the whole cell lysate samples.

### LDH assay

LDH activity in conditioned culture media was measured using a Lactic Dehydrogenase Based In Vitro Toxicology Assay Kit (Sigma) [53, 54, 56]. Each histogram represents data from at least three independent experiments performed with different cell populations.

### Statistics

Data analysis was performed on GraphPad Prisms Software (Graphpad Inc.). For comparison of the conjugation levels of SUMO2/3 or SUMO1, between time-matched two groups, paired Student’s *t*-test with a two-tail *P*-value was performed. For comparisons of the levels of FKBP8, FIS1, LC3-II, SENP1, SENP3, SQSTM, TOM20 or TBC1D17, and for analysis of the mitophagic levels per cell and SENP3 levels in whole cell lysate and subcellular fractions, between two groups, unpaired Student’s *t*-test with a two-tail *P*-value was performed. For comparison of multiple data sets, one-way analysis of variance followed by Sidak’s (for imaging data) or Tukey’s (for LDH data) multiple comparisons test was used.

## Supplementary information

Supplemental Figures and Material (full-length blots)

## Data Availability

The data underlying the findings of this study are available from the corresponding authors upon reasonable request.
