# Supplementary material for: SENP3-FIS1 axis promotes mitophagy and cell survival under hypoxia
Source: Cell Death Dis. 2024 Dec 5;15(12):881. doi: 10.1038/s41419-024-07271-8 (PMC11621581; doi:10.1038/s41419-024-07271-8)

Supplemental figures

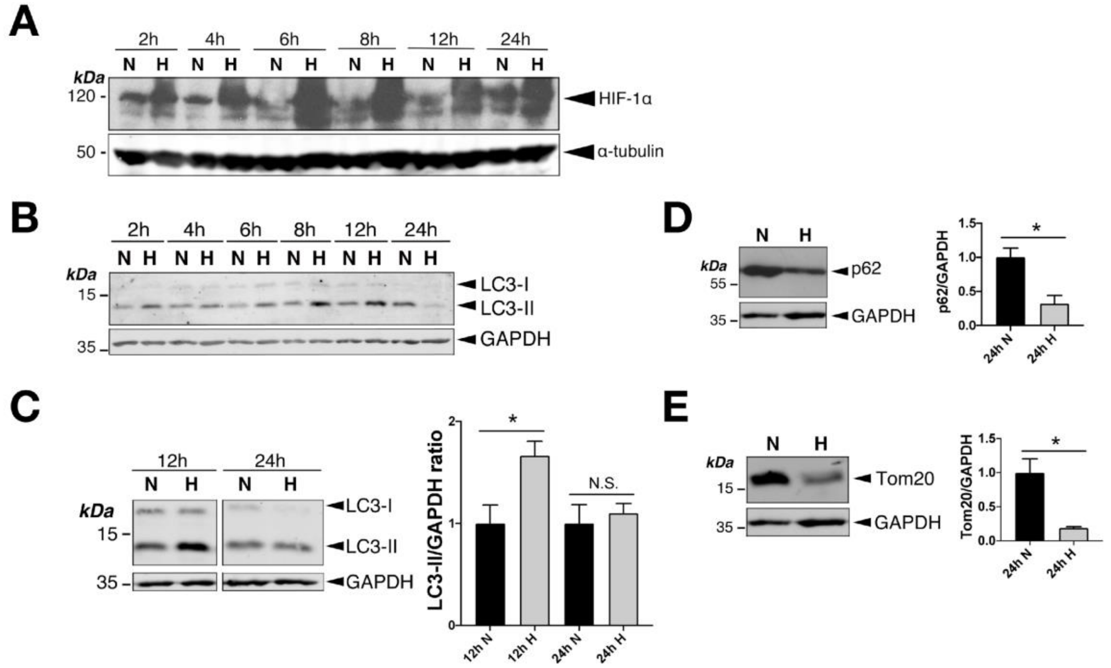

Fig. S1: Hypoxia-induced autophagy is a temporal process in HeLa cells. **A** HIF-1α Induction under Hypoxia in HeLa Cells. HeLa cells were exposed to normoxia or hypoxia (1% O_2) for 2 to 24 h. Whole cell lysate samples were prepared and blotted for HIF-1α as indicated. **B** Hypoxia induces changes in LC3-II levels. HeLa cells were exposed to 1% O_2 for 2, 4, 6, 8, 12, or 24 h. Hypoxia-induced LC3-II is detectable as early as 2h after cells exposed to 1% O_2 and become unnoticeable in cells exposed to 1% O_2 for 24 h. **C** LC3-II induction is time-dependent. HeLa cells were exposed to 1% O_2 for 12 or 24 h. Upper panel are representative blots showing LC3-II levels under indicated conditions, and Histogram in the right panel shows relative LC3-II level under indicated conditions (n=3 biological replicates; N.S., not statistically significant; *p<0.05; unpaired *t*-test). **D** Exposure of HeLa cells to hypoxia reduces cellular p62 levels. HeLa cells were exposed to normoxia or hypoxia (1% O_2) for 24 h. Whole cell lysate samples were prepared and blotted as indicated. Histogram in the right panel shows relative p62 levels in HeLa cells exposed to normoxia (N) or hypoxia (H) for 24 h (n=3 biological replicates; N.S.,

not statistically significant; *p<0.05; unpaired *t*-test). **E** Exposure of HeLa cells to hypoxia reduces cellular Tom20 levels. HeLa cells were exposed to normoxia or hypoxia (1% O_2) for 24 h. Whole cell lysate samples were prepared and blotted for Tom20 as indicated. Histogram in the right panel shows relative p62 levels in HeLa cells exposed to normoxia (N) or hypoxia (H) for 24 h (n=3 biological replicates; N.S., not statistically significant; *p<0.05; unpaired *t*-test).

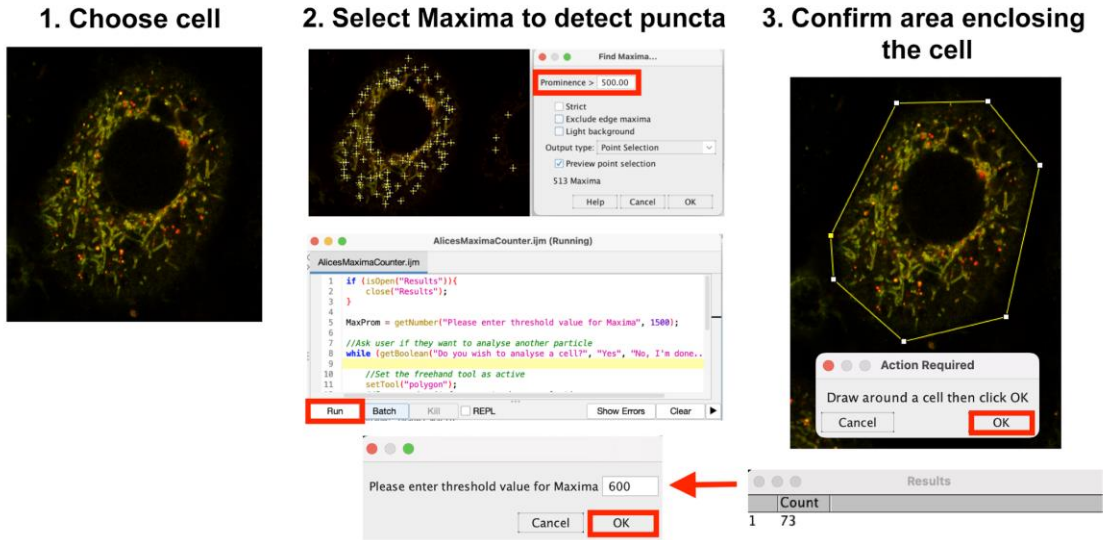

Fig. S2: Overview of Mitophagy Quantification indicated by Mito-pHfluorin in Fiji

Step 1: Open microscopy image and choose appropriate cell.

Step 2: In Fiji, select the red channel of the image. Open Process > Find Maxima. Toggle the Maxima up to the value 1500 and preview the red puncta with "Preview point selection" until the majority of puncta appear selected in the preview. Close the "Find Maxima" menu and run the counting program, entering the Maxima value chosen.

Step 3: Using the polygon selection tool, draw around the area enclosing the cell to count the red puncta.

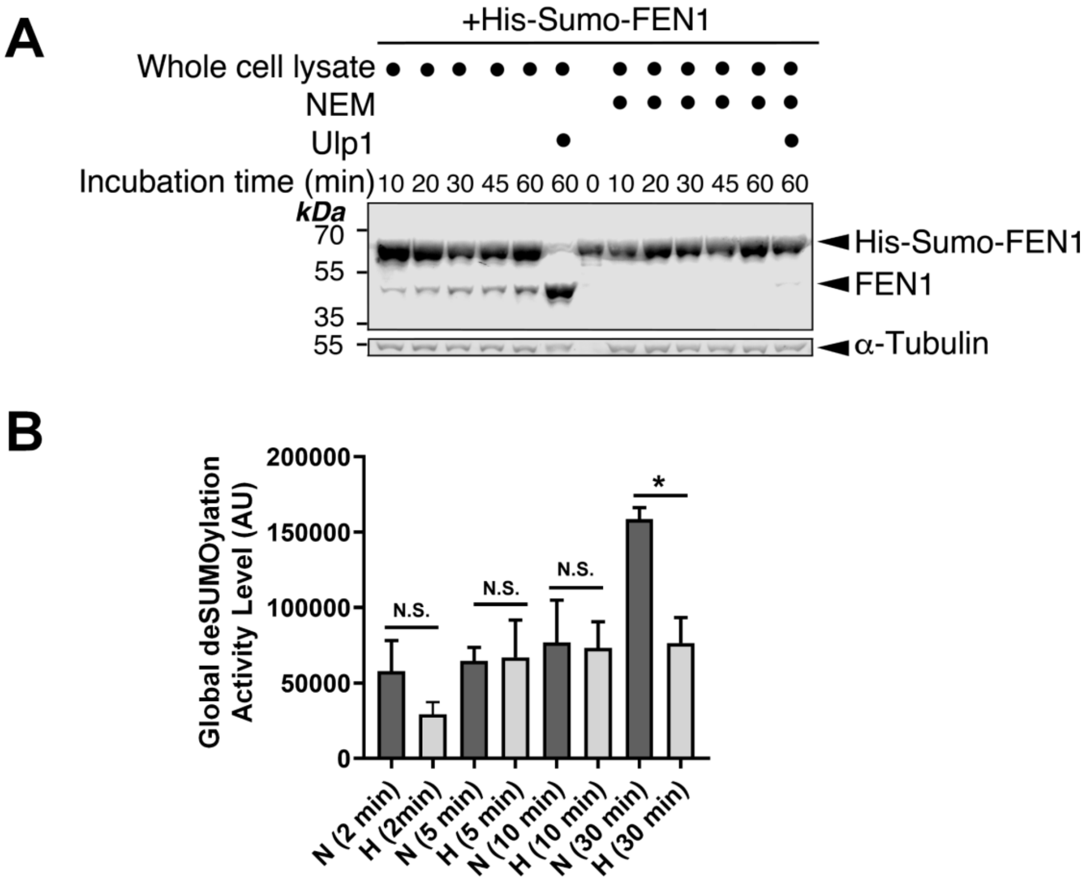

Fig. S3: Establishment of a novel assay for assessing global SUMO protease activities in cell/tissue lysate samples. **A** a SUMO protease assay was established using His-Sumo-FEN1 as a substrate. 1 µg of His-Sumo-FEN1 was added to 20 µl of HeLa cell whole cell lysate (approximately 1 µg/µl) and incubated at 30°C for varying durations. To inhibit deSUMOylation, NEM (10 mM) was added to some reactions. The addition of Ulp1 (0.8 µM) served as a positive control, demonstrating the removal of the His-Sumo tag from His-Sumo-FEN1. **B** Statistical comparison of global deSUMOylation activity levels in cell lysate samples prepared from HeLa cells exposed to normoxia and hypoxia for 24 h (as depicted in Figure 2; n=3, biological replicates; N.S., not statistically significant; * $p<0.05$; unpaired t-test).

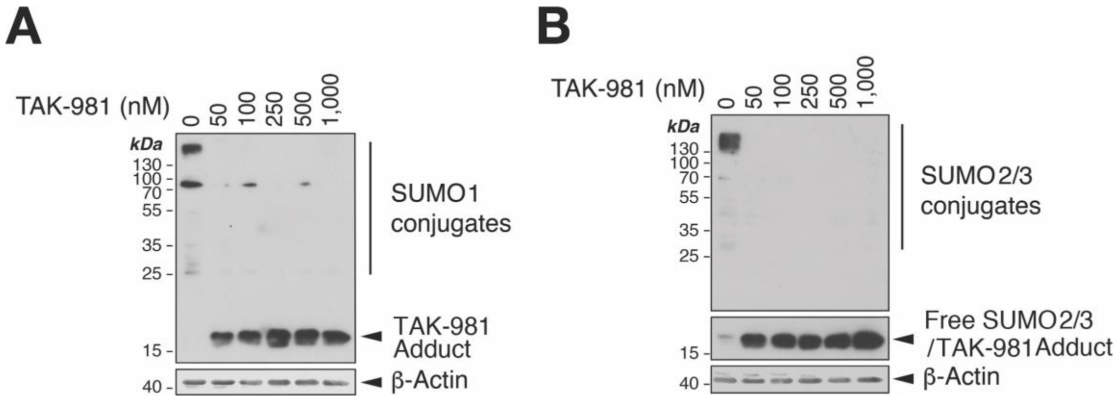

Fig. S4: TAK-981 Treatment inhibits SUMO1 (A) and SUMO2/3 (B) conjugation in HeLa cells. HeLa cells were treated with increasing concentrations of TAK-981 (0-1000 nM) for 4 h. Whole cell lysate samples were prepared blotted as indicated.

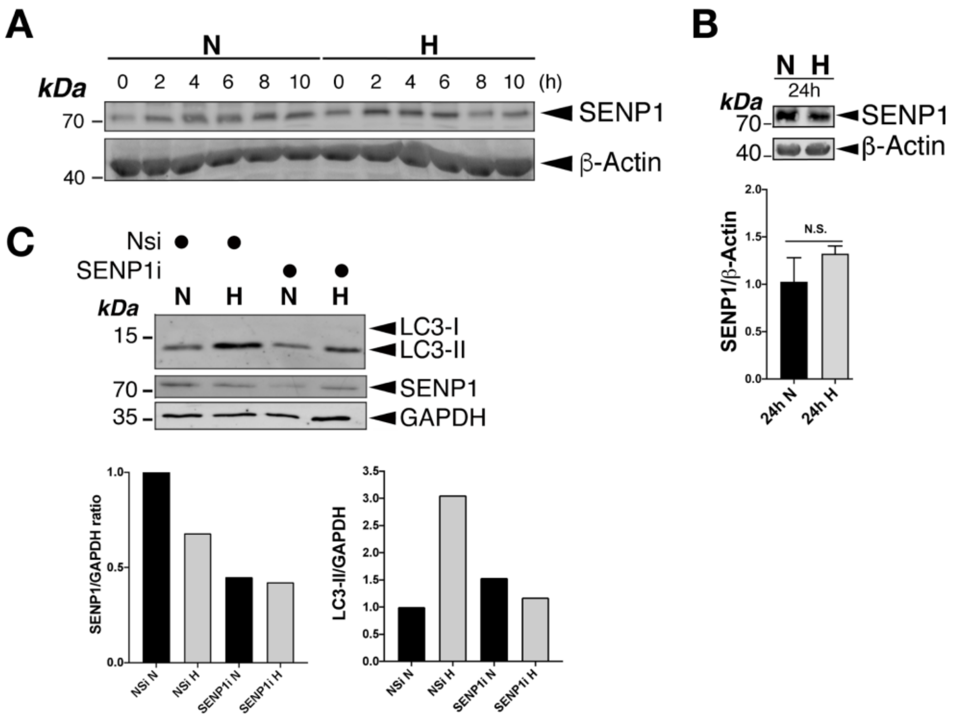

Fig. S5: Involvement of SENP1 in LC3-II induction by hypoxia in HeLa cells. **A** Hypoxia does not seem to result in apparent changes in SENP1 levels in HeLa cells exposed to 1%O_2 for 2~10 h. **B** Exposure of cells to hypoxia for 24 h does not result in changes in SENP1 levels. Histogram in the lower panel shows the relative levels of SENP1 in the indicated conditions (n=3 biological replicates;

N.S., non-significant; unpaired *t*-test). **C** RNAi-mediated SENP1 depletion appears to prevent LC3-II induction by hypoxia in HeLa cells. HeLa cells were transfected with Nsi or SENP1-specific siRNA (SENP1i). 48 h post-transfection the cells were exposed to normoxia or hypoxia (1% O_2) for 8 h. In (A)-(C) whole cell lysate samples were immunoblotted as indicated and quantitatively examined.

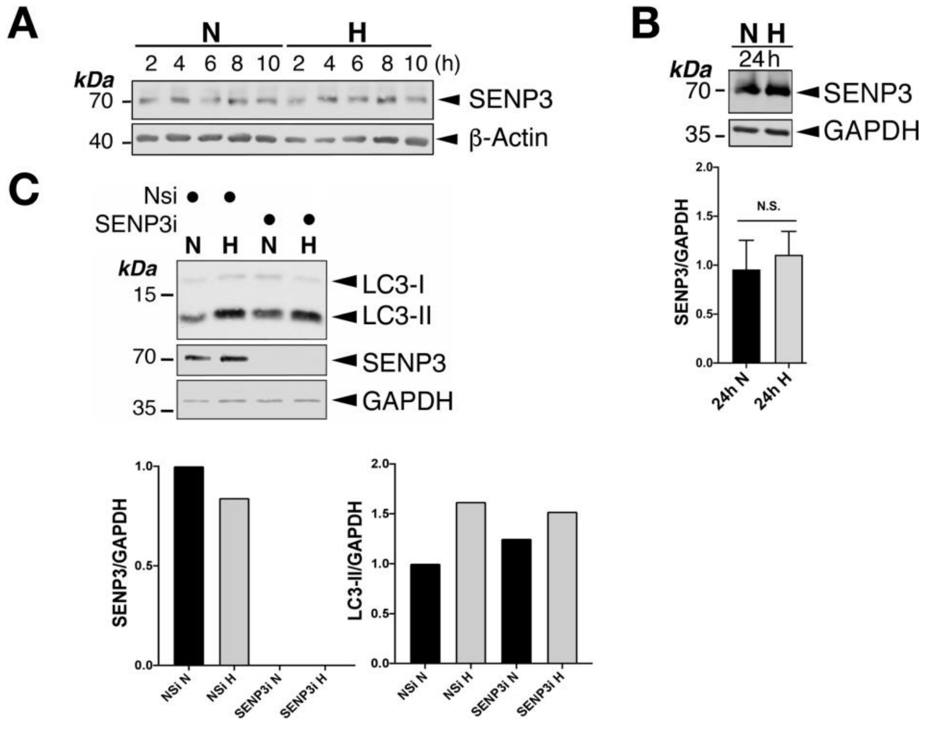

Fig. S6: No apparent Involvement of SENP3 in LC3-II induction by hypoxia in HeLa cells. **A** Hypoxia does not seem to result in apparent changes in SENP3 levels in HeLa cells exposed to 1%O_2 for 2~10 h. **B** Exposure of cells to hypoxia for 24 h does not result in changes in SENP3 levels. Histogram in the lower panel shows the relative levels of SENP3 in the indicated conditions (n=3 biological replicates; N.S., non-significant; unpaired *t*-test). **C** RNAi-mediated SENP3 depletion does not appear to affect LC3-II induction by hypoxia in HeLa cells. HeLa cells were transfected with Nsi or SENP3-specific siRNA (SENP3i). 48 h post-transfection the cells were exposed to normoxia or

hypoxia (1% O_2) for 8 h. In (A)-(C) whole cell lysate samples were immunoblotted as indicated and quantitatively examined.

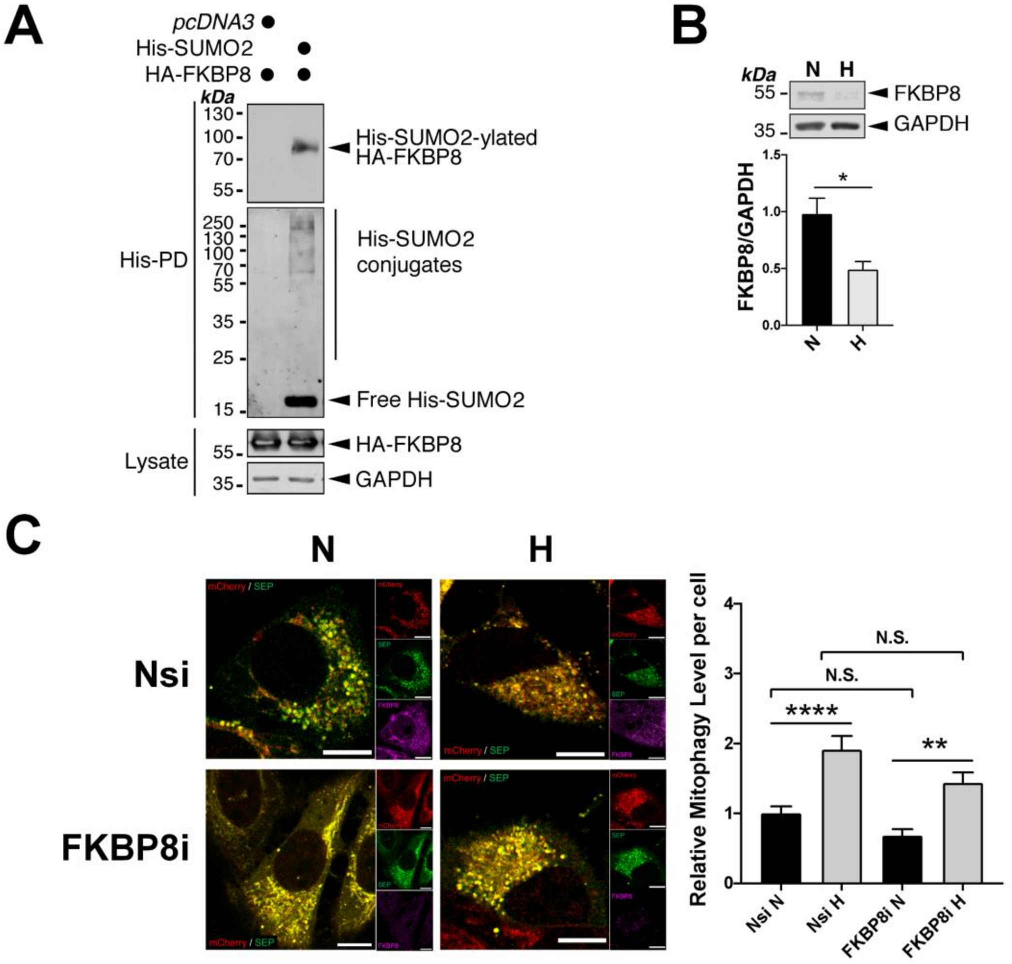

Fig. S7: Role of FKBP8 in hypoxia-induced mitophagy in HeLa cells. **A** FKBP8 is His-SUMO2-ylated in HEK293 cells. His-SUMO2 together with HA-FKBP8 were transfected into HEK293 cells for 48h. pcDNA3 was used as a control for His-SUMO2. His-PD and lysate samples were detected by immunoblotting for HA or GAPDH. **B** Exposure of HeLa cells to hypoxia reduces cellular FKBP8 levels. HeLa cells were exposed to normoxia or hypoxia (1% O_2) for 24 h. Whole cell lysate samples were prepared blotted as indicated. Histogram in the right panel shows relative levels of FKBP8 in HeLa cells exposed to N or H for 24 h. (n = 3, *p <0.05, unpaired *t*-test; representative of 3 experiments using independent cell populations). **C** RNAi-mediated FKBP8 depletion does not

affect mitophagy induced by hypoxia. HeLa cells expressing Mito-pHfluorin were transfected with Nsi or FKBP8-specific siRNA (50 nM). 48 h post-transfection the cells were exposed to normoxia or hypoxia (1% O_2) for 24 h, and the cells were analyzed 72 h post-transfection (Scale bar 10 μm). Histogram in the right panel shows relative mitophagy level per cell for cells exposed to N or H for 24 h (n = 34-51, N.S. non-significant; **p <0.01; ****p<0.0001; Ordinary one-way ANOVA followed by Sidak's multiple comparisons test).

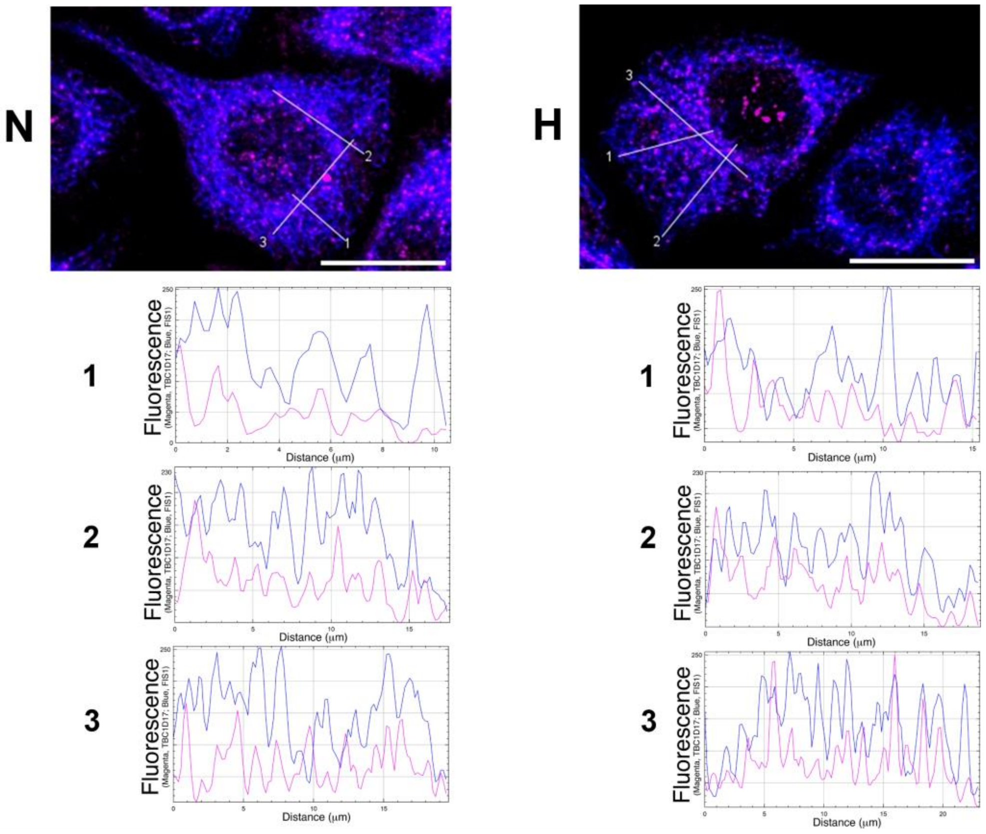

Fig. S8: Hypoxia does not appear to result in changes in the colocalisation between TBC1D17 and FIS1. HeLa cells were exposed to normoxia (N) or hypoxia (H; 1% O_2) for 24 h. Cells were fixed and stained for TBC1D17 (magenta) and FIS1 (blue) (Scale bar 20 μm). Relative fluorescence intensity of each channel as points along the white lines drawn randomly were shown in the lower panel graphs for N and H, respectively.

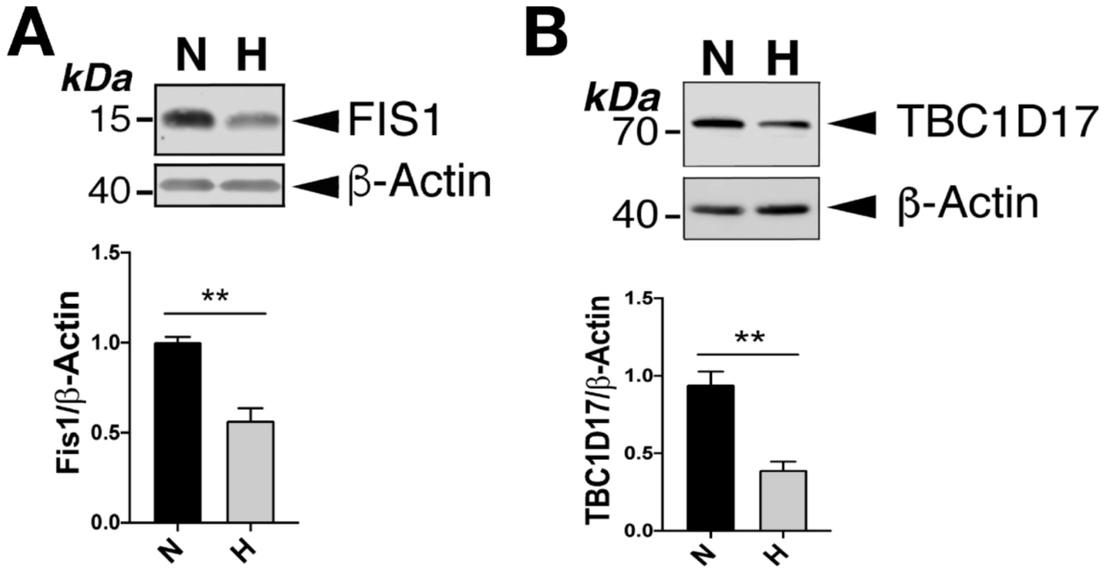

Fig. S9: Hypoxia results in decreased cellular levels of FIS1 and TBC1D17. HeLa cells were exposed to normoxia or hypoxia (1% O_2) for 24 h. Whole cell lysate samples were prepared and blotted as indicated. Histograms in the lower panels show relative levels of FIS1 (A, n = 3 biological replicates; *p <0.01, unpaired *t*-test; representative of 6 experiments using independent cell populations) or TBC1D17 (B, n = 3 biological replicates; **p<0.01; Unpaired *t*-test; representative of 3 experiments using independent cell populations).

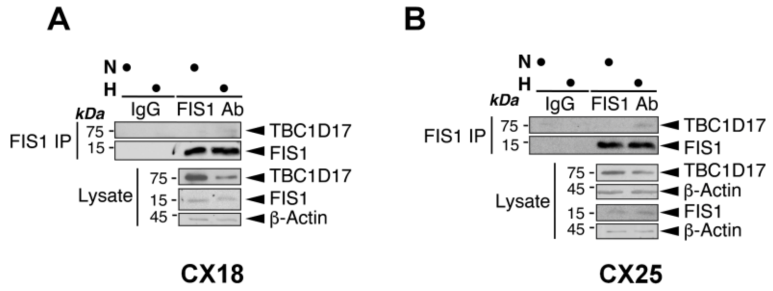

Fig. S10: Hypoxia induces FIS1-TBC1D17 complex formation in primary GSCs. GSCs (A, CX18 or B, CX25) were exposed to normoxia or hypoxia (1% O_2) for 24 h. FIS1 was enriched through IP. Lysate (input) and IP samples were immunoblotted as indicated.

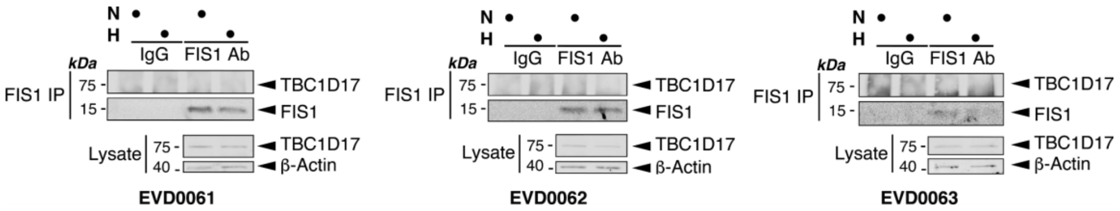

Fig. S11: Absence of FIS1-TBC1D17 complex formation in primary cell cultures derived from renal cancer patients. Primary renal cell cultures (EVD0061-63) were exposed to normoxia or hypoxia (1% O_2) for 24 h. FIS1 was enriched through IP. Lysate (input) and IP samples were immunoblotted as indicated.

Original blots

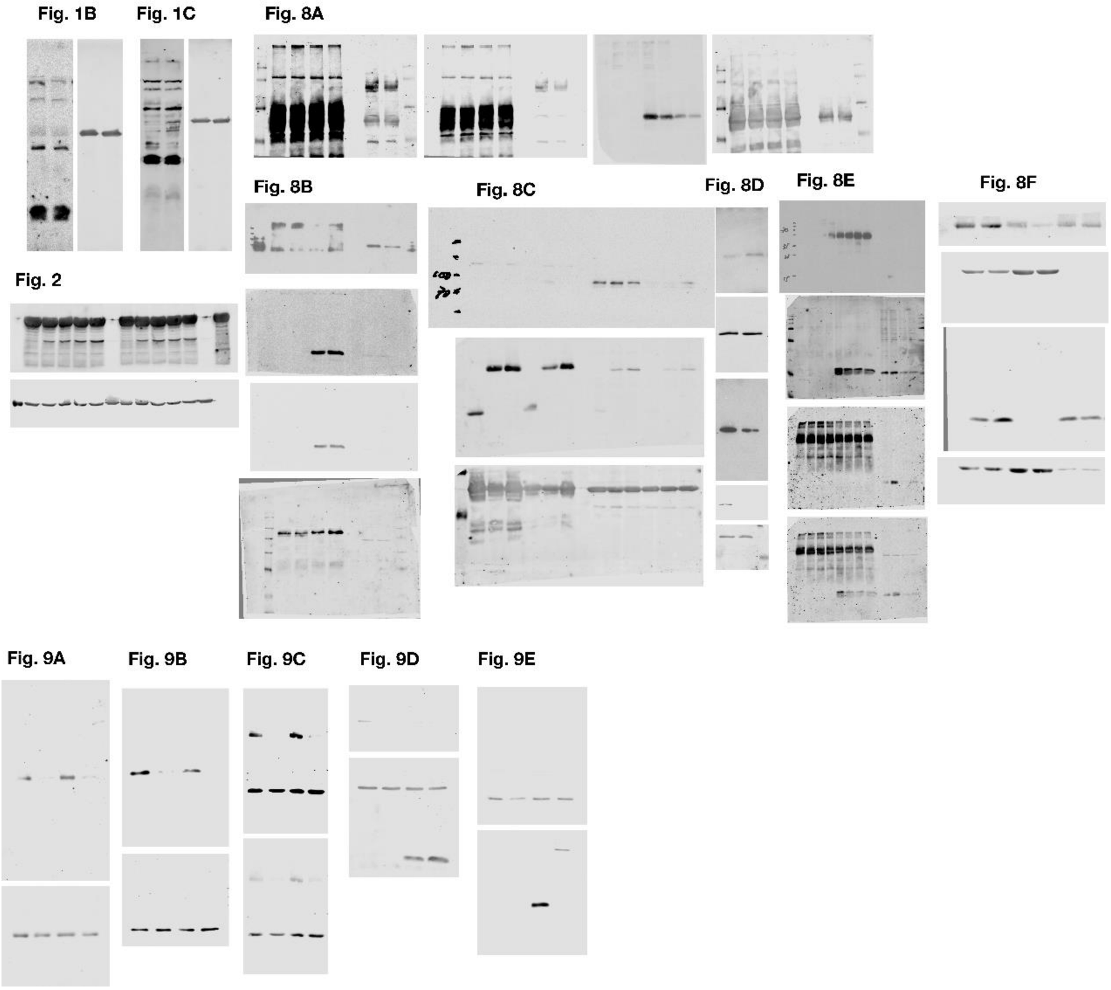

Supplement: Supplementary file 1 — Supplemental Figures and Material (full-length blots) [file 41419_2024_7271_MOESM1_ESM.pdf]
